# LINC01614 is a promising diagnostic and prognostic marker in HNSC linked to the tumor microenvironment and oncogenic function

**DOI:** 10.3389/fgene.2024.1337525

**Published:** 2024-04-09

**Authors:** Xiong Tian, Dali Hu, Na Wang, Lele Zhang, Xuequan Wang

**Affiliations:** ^1^ Key Laboratory of Minimally Invasive Techniques & Rapid Rehabilitation of Digestive System Tumor of Zhejiang Province, Linhai, China; ^2^ Department of Public Research Platform, Taizhou Hospital of Zhejiang Province Affiliated to Wenzhou Medical University, Linhai, China; ^3^ Department of Plastic Surgery, Taizhou Hospital of Zhejiang Province Affiliated to Wenzhou Medical University, Linhai, China; ^4^ Department of Clinical Laboratory, Taizhou Hospital of Zhejiang Province Affiliated to Wenzhou Medical University, Linhai, China; ^5^ Department of Radiation Oncology, Taizhou Hospital of Zhejiang Province Affiliated to Wenzhou Medical University, Linhai, Zhejiang, China

**Keywords:** pan-cancer, LINC01614, immune infiltration, prognosis, stemness, head and neck squamous cell carcinoma

## Abstract

**Background:**

Tumor initiation and metastasis influence tumor immune exclusion and immunosuppression. Long non-coding RNA (lncRNA) LINC01614 is associated with the prognosis and metastasis of several cancers. However, the relationship between LINC01614 and cancer immune infiltration and the biofunction of LINC01614 in head and neck squamous cell carcinoma (HNSC) remain unclear.

**Methods:**

The Genotype-Tissue Expression (GTEx) and The Cancer Genome Atlas (TCGA) datasets were used to analyze the expression difference and diagnostic value of LINC01614 in normal and tumor tissues. The correlation of pan-cancer prognosis and tumor stage of LINC01614 was analyzed based on the TCGA database. The pan-cancer association of LINC01614 expression with the tumor microenvironment (TME) including immune infiltration, expression of immune-related genes, and genomic instability parameters, was analyzed using the Spearman correlation method. The correlation between LINC01614 and tumor stemness evaluation indicators, RNA methylation-related genes, and drug resistance was also analyzed. The functional analysis of LINC01614 was performed using the clusterProfiler R package. The protein–protein interaction (PPI) network and ceRNA network of LINC01614 co-expressed genes and miRNA were constructed and visualized by STRING and Cytoscape, respectively. Finally, the cell location and influence of LINC01614 on cell proliferation and metastasis of HNSC cell lines were evaluated using FISH, CCK-8, wound-healing assay, and transwell assay.

**Results:**

LINC01614 was found to be overexpressed in 23 cancers and showed a highly sensitive prediction value in nine cancers (AUC >0.85). LINC01614 dysregulation was associated with tumor stage in 12 cancers and significantly influenced the survival outcomes of 26 cancer types, with only Lymphoid Neoplasm Diffuse Large B-cell Lymphoma (DLBC), uterine corpus endometrial carcinoma (UCEC), and bladder urothelial carcinoma (BLCA) showing a benign influence. LINC01614 was also associated with immune cell infiltration, tumor heterogeneity, cancer stemness, RNA methylation modification, and drug resistance. The potential biological function of LINC01614 was verified in HNSC, and it was found to play important roles in proliferation, immune infiltration, immunotherapy response, and metastasis of HNSC.

**Conclusion:**

LINC01614 may serve as a cancer diagnosis and prognosis biomarker and an immunotherapy target for specific cancers.

## 1 Introduction

Cancers with the highest global morbidity and mortality rates have inflicted substantial health and economic burdens on the society (Sung et al., 2021). Despite advances in diagnosis and treatment, survival rates for most cancers remain low due to drug resistance, side effects, or undesired toxicities.

Therefore, there is an urgent need to identify alternative and sensitive tumor biomarkers that can suppress the proliferation and progression of cancer cells for improved cancer diagnosis and treatment (Li and Song, 2020). Immunotherapies, which reactivate the adaptive and innate immune systems, have emerged as an alternative to classical anticancer treatments and have exerted potent antitumor immune responses. Inhibitors of programmed death 1 (PD-1), programmed death ligand 1 (PD-L1), and cytotoxic T lymphocyte-associated antigen 4 (CTLA4) have demonstrated antitumor effects in malignant melanoma and non-small-cell lung cancer (Wang et al., 2022a). Immunotherapy has been shown to be beneficial for tumor patients. Biomarkers associated with immune checkpoint blockade (ICB) therapy have become a promising strategy in cancer treatment (Bagchi et al., 2021). Therefore, the identification of potential targets for immunotherapy is in urgent need. Long noncoding RNAs (lncRNAs) are noncoding transcripts longer than 200 nucleotides, and aberrant expression of particular lncRNAs has been proven to generally associate with proliferation, apoptosis, metabolism, immune escape, and migration or invasion of cancer cells (Huarte, 2015). It is worth noting that some lncRNAs can serve as early diagnosis biomarkers or prognosis parameters in different human carcinomas (de Santiago et al., 2021). Emerging evidence suggests a compelling correlation between LINC01614 expression and prognostic outcomes of lung cancer (Liu et al., 2022), gastric cancer (Chen et al., 2021), and ESCA prognosis (Wei et al., 2023). LINC01614 was relevant to the HR+/HER2 + breast cancer molecular subtype and accelerated breast cancer cell proliferation and migration by TGF-β and focal adhesion kinase (FAK) signaling (Vishnubalaji et al., 2019). In gastric cancer, LINC01614 was related to ferroptosis and could be used as a potential prognosis factor (Cai et al., 2022). The carcinogenic function of LINC01614 through the ceRNA mechanism has also been reported. For instance, LINC01614 upregulated FOXP1 by binding miR-217 and promoted LUAD cancer development (Liu et al., 2018a). In osteosarcoma, LINC01614 accelerated SNX3 expression by binding miR-520a-3p, further leading to cancer progression (Cai et al., 2021). Wang et al. (2020a) demonstrated that LINC01614 is a highly cancer-associated oncogene and can serve as a prognostic biomarker for multiple types of tumors (Wang et al., 2020a). LncRNAs have been reported to regulate distinct immune cell functions in the tumor immune microenvironment (TIME) through diverse biological processes (Park et al., 2022). However, the influence of LINC01614 on the TME remains to be studied.

Through pan-cancer data mining, our study revealed that LINC01614 was consistently upregulated and could serve as a diagnostic or prognostic marker in most cancer types. LINC01614 expression was associated with immune cell infiltration, cancer stemness, drug resistance, genomic instability, etc. Specifically, in head and neck squamous cell carcinoma (HNSC), LINC01614 was predominantly located in the cytoplasm and appeared to play a crucial role in promoting cellular proliferation, immune infiltration, immunotherapy response, and metastasis. Overall, our study highlighted the potential for LINC01614 as a tumor diagnostic and prognostic marker, particularly in the context of HNSC.

## 2 Materials and methods

### 2.1 Pan-cancer expression and diagnosis analysis of LINC01614

RNA-seq and clinical data of 9,125 patients for 33 cancers were extracted from the Genotype-Tissue Expression (GTEx) and The Cancer Genome Atlas (TCGA) databases through the UCSC-Xena platform (https://toil.xenahubs.net). Differences in LINC01614 expression between normal and tumor samples were evaluated across a combined GTEx and TCGA cohort, a separate TCGA cohort, and within TCGA paired samples cohort (where tumor and normal tissues were obtained from the same patients). The evaluation was conducted using the Wilcoxon rank-sum test, with a *p*-value of less than 0.05 indicating statistical significance. After the molecular subtype data for TCGA samples were extracted from the “TCGAbiolinks” R package, the expression differences of LINC01614 across various molecular subtypes or stages were analyzed using the Kruskal–Wallis test. The potential diagnostic value of LINC01614 in different cancers was examined using receiver operating characteristic (ROC) analysis based on TCGA and GTEx databases. The area under the curve (AUC) > 0.85 was considered of high diagnostic value.

### 2.2 The pan-cancer prognosis analysis of LINC01614 expression

The survival information including progression-free interval (PFI), progression-free survival (PFS), disease-specific survival (DSS), and disease-free interval (DFI) was extracted from Liu et al. (2018b). After patients from each cancer were grouped into high- and low-LINC01614 expression groups using the “survminer” package based on different clinical outcome endpoints, the prognostic value of the LINC01614 expression level was then investigated using Cox proportional hazards regression, the Kaplan–Meier curve method, and the log-rank test. The Cox regression results were presented using the “forestplot” R packages, with a *p*-value <0.05 regarded as indicating statistical significance.

### 2.3 LINC01614 expression and immune correlation analysis in pan-cancer

The immune infiltration estimations of TCGA pan-cancer patients calculated by TIMER, EPIC, QUANTISEQ, XCELL, MCPCOUNTER, and CIBERSORT-ABS methods were downloaded from the TIMER2.0 database (http://timer.cistrome.org/) (Li et al., 2020). The correlation of LINC01614 expression and these immune infiltration estimations for each cancer type was analyzed using Pearson’s correlation analysis. The pan-cancer coexpression of LINC01614 with immune-related genes including immune inhibitors, immune stimulators, chemokines and chemokine receptors, and major histocompatibility complex (MHC)-related genes was also analyzed using Spearman’s correlation method. All these correlation analyses were visualized using the “ComplexHeatmap” package.

### 2.4 Correlation between LINC01614 expression and genomic instability

Tumor heterogeneity presents an ongoing challenge for tumor progression and treatment by affecting tumor growth, invasion, and drug sensitivity in many solid tumor types (Pang et al., 2020). Heterogeneity biomarkers including tumor mutational burden (TMB), microsatellite instability (MSI), mutant–allele tumor heterogeneity (MATH), homologous recombination deficiency (HRD), purity, ploidy, neoantigen, and loss of heterozygosity (LOH) were analyzed for the correlation with LINC01614 expression. TMB and MSI were calculated using the “maftools” R package by using the simple nucleotide variation dataset for all TCGA samples downloaded from Genomic Data Commons (GDC) (https://portal.gdc.cancer.gov/). MSI data were extracted from Bonneville et al. (2017). Other genomic instability evaluation values including HRD, LOH, number of neoantigens, purity, and ploidy were obtained from Huang and Fu (2019).

### 2.5 Correlation analysis of LINC01614 expression and tumor stemness

Characterized by self-renewal and tumor heterogeneity, tumor stem cells play a significant role in tumor proliferation, metastasis recurrence, and prognosis. DNA methylation-based stemness scores (mDNAss) and mRNA expression-based stemness scores (mRNAss) describing six stemness indexes (mRNAsi, mDNAsi, EREG−mRNAsi, EREG-mDNAsi, DMPsi, and ENHsi) of 33 tumors were calculated using a one-class logistic regression algorithm (Malta et al., 2018). Subsequently, Spearman’s correlation analysis was performed to analyze the correlation between LINC01614 expression and six tumor stemness indicators.

### 2.6 RNA methylation-related genes and LINC01614 co-expression analysis

RNA methylation is a reversible post-translational process of catalytic transferring of methyl groups to different RNA types by a variety of RNA-methyltransferases (Song et al., 2021). Studies have shown that RNA methylation is an essential epigenetic regulatory pathway involved in tumorigenesis, progress, and prognosis of cancers (Han et al., 2021). The coexpression relationship between LINC01614 expression and different RNA methylation regulators, including nl-methyladenosine (m1A), n6-methyladenosine (m6A), and 5-methylcytosine (m5C), in pan-cancer tumor samples was analyzed using the Spearman correlation method.

### 2.7 Functional enrichment analysis of LINC01614 in HNSC

After 50 hallmark gene set collections were downloaded from the MSigDB v7.1 database (Liberzon et al., 2015) (http://software.broadinstitute.org/gsea/msigdb/index.jsp), gene set enrichment analysis (GSEA) based on LINC01614 expression values was performed using the R package “clusterProfiler”. The *p*-values were adjusted using the “BH” method, and an adjusted *p*-value < 0.05 was considered significant. After finding that LINC01614 was enriched with metastasis-related gene sets, pathologic N (N0, N1, N2, and N3)-related genes selected using the Kruskal–Wallis test were defined as metastasis-associated genes. LINC01614-coexpressed metastasis-associated genes were then selected by Pearson’s correlation analysis. The functional enrichments were then analyzed by Gene Ontology (GO) and Kyoto Encyclopedia of Genes and Genomes (KEGG) pathway enrichment analyses using the “clusterProfiler” package. The interaction between each co-expressed protein-coding genes was analyzed using the STRING database (http://www.string-db.org/), with the minimum interaction score being 0.4 (medium confidence). The CytoNCA plug-in (Version 2.1.6) in Cytoscape software (version 3.4.0, http://chianti.ucsd.edu/cytoscape-3.4.0/) was used to calculate and visualize node connectivity in the protein–protein interaction network.

### 2.8 LINC01614-associated ceRNA construction

The miRNA with potential binding ability for the 3′UTR (untranslated region) of LINC01614 was predicted by miRWalk 3.0 (http://mirwalk.umm.uni-heidelberg.de/search_genes/) with binding probability >95% as the threshold. The experimentally validated miRNA–mRNA pairs for LINC01614 were screened using the miRTarBase database. The selected LINC01614–miRNA relationship pairs were selected by the miRanda software (v3.3a) with suitable parameters (score > = 140 and energy ≤ −20). LINC01614 positively co-expressed mRNAs (correlation coefficient >0.4 and *p* < 0.05) and miRNA that both bind with LINC01614, and these mRNAs were constructed as an LINC01614–miRNA–mRNA–ceRNA network by Cytoscape software.

### 2.9 Drug sensitivity analysis

Drug sensitivity analysis of LINC01614 expression across pan-cancer samples was estimated using the “oncoPredict” package based on Genomics of Drug sensitivity in Cancer (GDSC)-V2 data (https://www.cancerrxgene.org/downloads/anova) containing 198 anticancer compounds and 135,242 drug reaction inhibitory concentration 50% (IC_50_) values and TCGA pan-cancer expression values. Drugs that are significantly influenced by LINC01614 in HNSC were selected for visualization. The correlation of LINC01614 expression and drug activity was assessed in total cancer samples and each cancer sample. *p* < 0.05 was used as the threshold of significance.

### 2.10 Cell culture and RNA interference

Human laryngeal cancer cells Tu212 and Tu686 were obtained from the cell bank of the Chinese Academy of Sciences (Shanghai, China). These cells were cultured in IMDM supplemented with 10% fetal bovine serum. Incubation was carried out at 37°C in a 5% CO_2_ environment. The targeting LINC01614 shRNA sequence and negative control were synthesized by GENEWIZ (South Plainfield, NJ). These sequences are as follows:

shRNA-1: 5’ -GGA​ATA​GGC​TCT​CTT​TCT​CTT​AA-3’;

shRNA-2: 5′-GCC​AAA​GTC​AAT​ATC​TCA​AAG-3’; negative control:

5′-CAA​CAA​GAT​GAA​GAG​CAC​CAA​CTC​GAG​TTG​GTG​CTC​TTC​ATC​TTG​TTG​TTT​TTG-3’.

The synthesized shRNA was cloned into the lentiviral vector pLVshRNA-EGFP (2A) Puro and cotransfected into 293T cells with psPAX2 and pMD2G plasmids by Lipofectamine^TM^ 3000 (Invitrogen, CA, United States) to generate lentiviral particles. The collected lentiviral particles, along with 8 mg/mL of polybrene, were used to perform a coinfection of TU212 and TU686 cancer cells.

### 2.11 RNA extraction and qPCR analysis

After 48 h of lentiviral infection, the cells were harvested, and the TRIzol reagent (Thermo Fisher Scientific, MA, United States) was used to extract the total RNA, following the manufacturer’s protocol. The quality and quantity of the extracted RNA were assessed using a spectrophotometer (Thermo Scientific, NanoDrop One). The extracted RNA samples were then subjected to reverse transcription to synthesize cDNA using the PrimeScript RT reagent Kit from Takara (Japan). The quantitative PCR (qPCR) was performed using the SYBR Green Master Mix from Takara (Japan) to quantify the relative expression of LINC01614. The expression of LINC01614 was normalized to the relative expression of GAPDH, which served as an internal control. The primer sequences are as follows:

LINC01614-F: 5′- AAC​CAA​GAG​CGA​AGC​CAA​GA-3’;

LINC01614-R: 5’ -GCT​TGG​ACA​CAG​ACC​CTA​GC-3’;

GAPDH-F: 5′- CTG​GGC​TAC​ACT​GAG​CAC​C-3’;

GAPDH-R: 5’ -AAG​TGG​TCG​TTG​AGG​GCA​ATG-3’.

### 2.12 CCK-8 assay

To assess the effect of LINC01614 knockdown on cell viability, Tu212 and Tu686 cells were plated at a density of 1.5 * 10^3 cells/well in a 96-well plate and incubated at 37°C. At specific time points, to each well was added 10 µL of CCK-8 reagent and incubated for 1 h. The absorbance of the samples was then measured at 450 nm using an absorption spectrophotometer.

### 2.13 Wound healing and invasion analysis

After cells reached almost 90% confluence in a six-well plate, the migration capacity was analyzed using wound healing assay. A 100-μL pipette tip was used to create scratches in the confluent monolayer. At 0 h and 48 h, the plates were evaluated and imaged by microscopy. For invasion assay, the bottom of the upper chamber was lined with a 1:8 dilution of Matrigel (BD, Biosciences), and the cells were inoculated in the upper chamber of the medium which contained 1% FBS at a density of 1*10^5. Transwells were placed in wells containing 20% FBS medium. Cells were fixed in 10% formalin on transwell membranes. The invaded cells were recorded under a ×10 magnification microscope in 5–10 random fields of view per well and quantified by ImageJ software.

### 2.14 Fluorescent *in situ* hybridization (FISH)

The LINC01614 probe was purchased from GenePharma (Shanghai, China). Cells were plated at a density of 10,000 cells per well in a 24-well plate with coverslips and incubated overnight. After fixing the cells with 4% paraformaldehyde, they were permeabilized using 0.1% Triton X-100. The pre-hybridization buffer was then removed, and the cells were subjected to overnight hybridization with the LINC01614 probe. Following hybridization, the cells were washed with SCC buffer. DAPI staining was performed to visualize the nuclei. Fluorescence testing was carried out using laser scanning confocal microscopy (Zeiss LSM800) to examine the expression of LINC01614 within the cells.

### 2.15 Statistical analysis

The statistical analyses were performed using R software (version 4.0.2) throughout the study. The independent sample t-test was conducted to quantify the analysis of variables. A *p*-value <0.05 was regarded as significant for all analyses, unless otherwise noted.

## 3 Results

### 3.1 LINC01614 expression was upregulated in most cancer types

**FIGURE 1:**
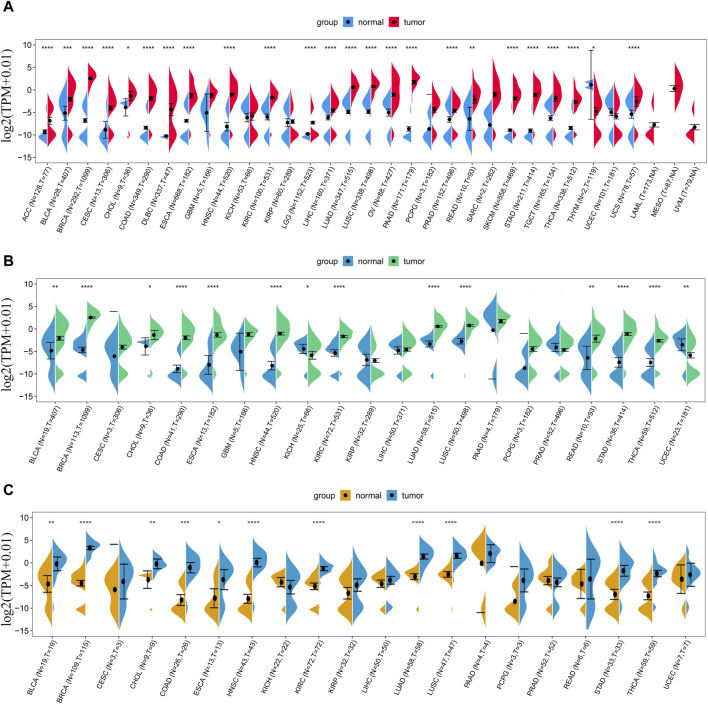
Expression difference analysis of LINC01614 in pan-cancer. **(A)** The expression levels of LINC01614 across 33 types of cancer tissues from TCGA and their corresponding normal tissues from GTEx and TCGA were analyzed. **(B)** LINC01614 expression analysis was performed in 21 tumors and corresponding normal tissues from the TCGA database. **(C)** Expression levels of LINC01614 were compared between tumor and paired adjacent non-cancerous tissues in 20 tumors from the TCGA database. Statistical significance was determined using the Wilcoxon rank sum test, and the results were denoted as follows: *p < 0.05, **p < 0.01, ***p < 0.001, and ****p < 0.0001.

We first analyzed the expression level of LINC01614 in 33 types of tumors and adjacent normal tissues from TCGA and GTEx databases. LINC01614 was significantly upregulated in 23 cancer types [adrenocortical carcinoma (ACC), bladder urothelial carcinoma (BLCA), breast invasive carcinoma (BRCA), cervical and endocervical cancer (CESC), cholangiocarcinoma (CHOL), colon adenocarcinoma (COAD), DLBC, esophageal carcinoma (ESCA), HNSC, kidney renal clear cell carcinoma (KIRC), brain lower-grade glioma (LGG), liver hepatocellular carcinoma (LIHC), lung adenocarcinoma (LUAD), lung squamous cell carcinoma (LUSC), ovarian serous cystadenocarcinoma (OV), pancreatic adenocarcinoma (PAAD), prostate adenocarcinoma (PRAD), rectum adenocarcinoma (READ), skin cutaneous melanoma (SKCM), stomach adenocarcinoma (STAD), testicular germ cell tumor (TGCT), thyroid carcinoma (THCA), and uterine carcinosarcoma (UCS)] but downregulated only in thymoma (THYM) (Figure 1A). Through TCGA database analysis, LINC01614 was significantly overexpressed in THCA, STAD, READ, LUSC, LUAD, KIRC, HNSC, ESCA, COAD, CHOL, BRCA, and BLCA cancers and underexpressed in uterine corpus endometrial carcinoma (UCEC) and kidney chromophobe (KICH) cancers (Figure 1B). The paired sample expression analysis revealed that LINC01614 was significantly elevated in BLCA, BRCA, CHOL, COAD, ESCA, HNSC, KIRC, LUAD, LUSC, STAD, and THCA cancers (Figure 1C). The above different expression analyses suggested that LINC01614 was significantly dysregulated and mainly upregulated in most cancers.

### 3.2 LINC01614 correlated with tumor stage, molecular subtypes, and tumor diagnosis in multiple cancers

**FIGURE 2:**
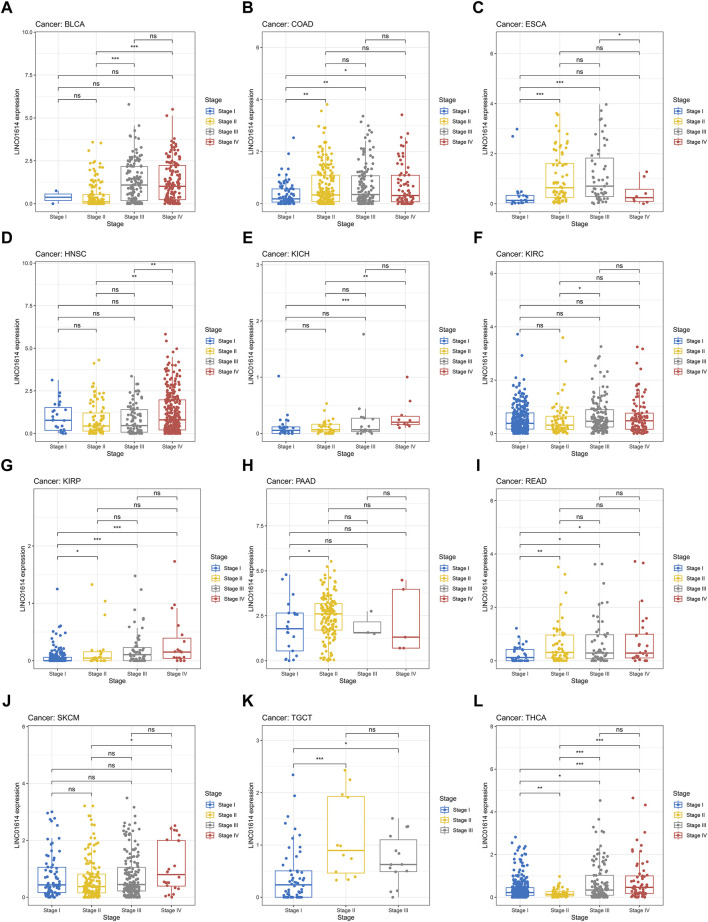
Significant correlations of LINC01614 expression with tumor stages. **(A)** BLCA, **(B)** COAD, **(C)** ESCA, **(D)** HNSC, **(E)** KICH, **(F)** KIRC, **(G)** KIRP, **(H)** PAAD, **(I)** READ, **(J)** SKCM, **(K)** TGCT, and **(L)** THCA(*p < 0.05, **p < 0.01, ***p < 0.001, and ****p < 0.0001).

**FIGURE 3:**
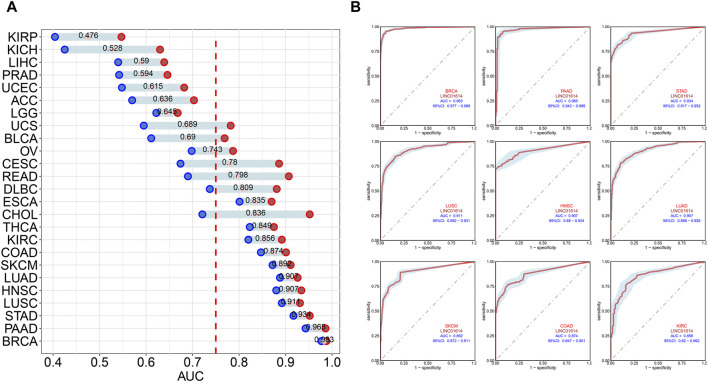
Diagnostic assessment of LINC01614 in pan-cancer by ROC analysis. **(A)** AUC values of ROC analysis for LINC01614 in diagnosing tumor and normal tissues across various types of cancer. **(B)** ROC curves for the top nine cancers diagnosed by LINC01614 with AUC >0.85, including BRCA, PAAD, STAD, SKCM, LUSC, LUAD, HNSC, COAD, and KIRC.

Correlation analysis of LINC01614 and tumor stage illustrated that LINC01614 expression was significantly correlated with the tumor stage of 12 human tumors (Figure 2A–L). LINC01614 expression was significantly increased in stage IV compared to stage I tumors in COAD, KICH, kidney renal papillary cell carcinoma (KIRP), READ, and THCA (Figure 2B, E, G, I, L). Significant upregulation was also found in stage IV compared to stage II tumors of BLCA, HNSC, KICH, SKCM, and THCA (Figure 2A, D, E, J, L); in stage II compared to stage I tumors of COAD, ESCA, KIRP, PAAD, READ, TGCT, and THCA (Figure 2B, C, G, H, I, K, L); in stage III compared to stage I tumors of COAD, ESCA, KIRP, READ, TGCT, and THCA (Figure 2B, C, G, I, K, L); in stage IV compared to stage III tumors of ESCA (Figure 2C); or in stage III compared to stage II tumors of BLCA, KIRC, and THCA (Figure 2A, F, L). In addition, we have conducted an examination of LINC01614 expression patterns across different molecular subtypes in a variety of cancers. Our findings indicate significant variability in LINC01614 expression among the molecular subtypes of seven cancers, namely, COAD, ESCA, HNSC, KIRP, LGG, LUSC, and STAD (Supplementary Figure S1). These significant differences encouraged our speculation that LINC01614 might be a tumor staging or progression influential factor, and a high level of LINC01614 might lead to an advanced tumor stage. This influence was also shown to be tumor-specific and may be more conducive to personalized treatment regimens. The diagnostic role of LINC01614 expression in distinguishing normal and tumor tissues was assessed using ROC analysis, which showed significant AUC values (AUC > 0.75) in 15 different cancers (Figure 3A). Among these, the top nine cancers displayed AUC values above 0.85, namely, BRCA (AUC = 0.983), PAAD (AUC = 0.965), STAD (AUC = 0.934), LUSC (AUC = 0.911), HNSC (AUC = 0.907), LUAD (AUC = 0.907), SKCM (AUC = 0.892), COAD (AUC = 0.874), and KIRC (AUC = 0.856). These findings suggest that LINC01614 might serve as an important diagnostic biomarker in these specific tumor types.

### 3.3 LINC01614 acted as an unfavorable prognosis biomarker in multiple cancers

**FIGURE 4:**
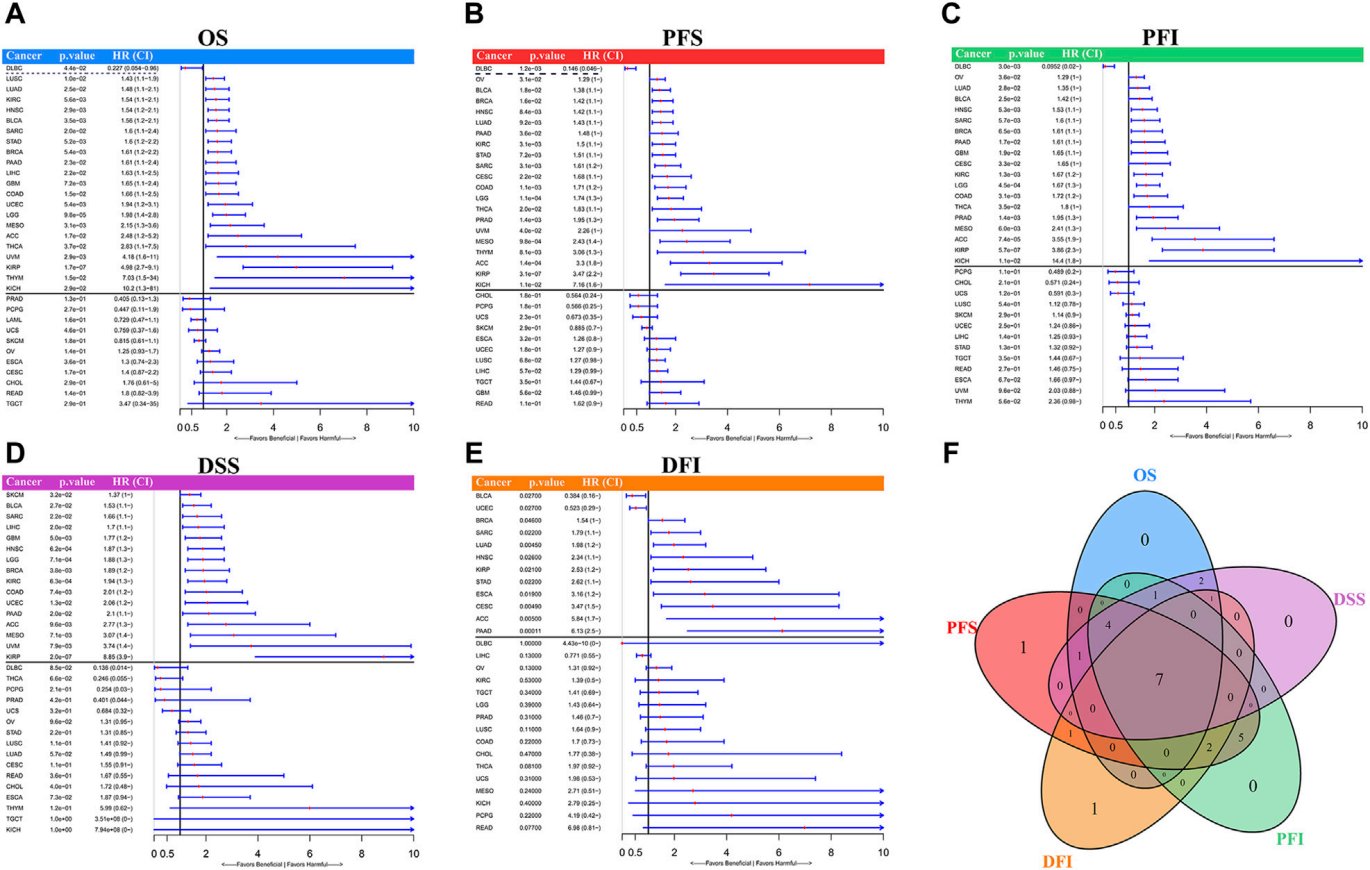
Univariate Cox regression for prognosis of LINC01614 expression in TCGA pan-cancer. The forest plots display the results of univariate Cox regression for LINC01614 expression in pan-cancer, including **(A)** OS, **(B)** PFS, **(C)** PFI, **(D)** DSS, and **(E)** DFI. **(F)** Venn plot illustrates the number of significant influences that overlap among different survival endpoints in pan-cancer.

Univariate Cox regression results assessing the prognostic value of LINC01614 expression levels indicated that high levels of LINC01614 predict short overall survival (OS) for 21 cancer types and predict long OS only for DLBC patients (Figure 4A). The PFS analysis revealed that LINC01614 acts as a risk factor for 23 cancer types and a protective factor for DLBC (Figure 4B). High levels of LINC01614 also predict poor DSS for 16 cancer types (Figure 4D), poor DFI for 10 cancers, and poor PFI for 18 cancers, whereas for DFI of BLCA and UCEC patients (Figure 4E) or for PFI of DLBC patients, LINC01614 played a protective role (Figure 4C). As shown by the Venn plot, LINC01614 expression has a significant influence on all five defined outcomes across seven types of cancer: ACC, BLCA, BRCA, HNSC, KIRP, PAAD, and SARC. Notably, it only demonstrated a favorable role in the DFI outcome for BLCA. The survival plots of above seven significant cancers are listed in the Kaplan–Meier’s survival curves (Supplementary Figure S2). A total of four cancers, namely, COAD, KIRC, LGG, and MESO, showed a significant relationship between LINC01614 expression and survival outcomes of OS, PFS, PFI, and DSS, except DFI (Figure 4F). These results suggested that LINC01614 expression had a significant influence in 11 cancers for more than four different survival endpoints.

### 3.4 Relationship between LINC01614 expression and immune cell infiltration in pan-cancer

**FIGURE 5:**
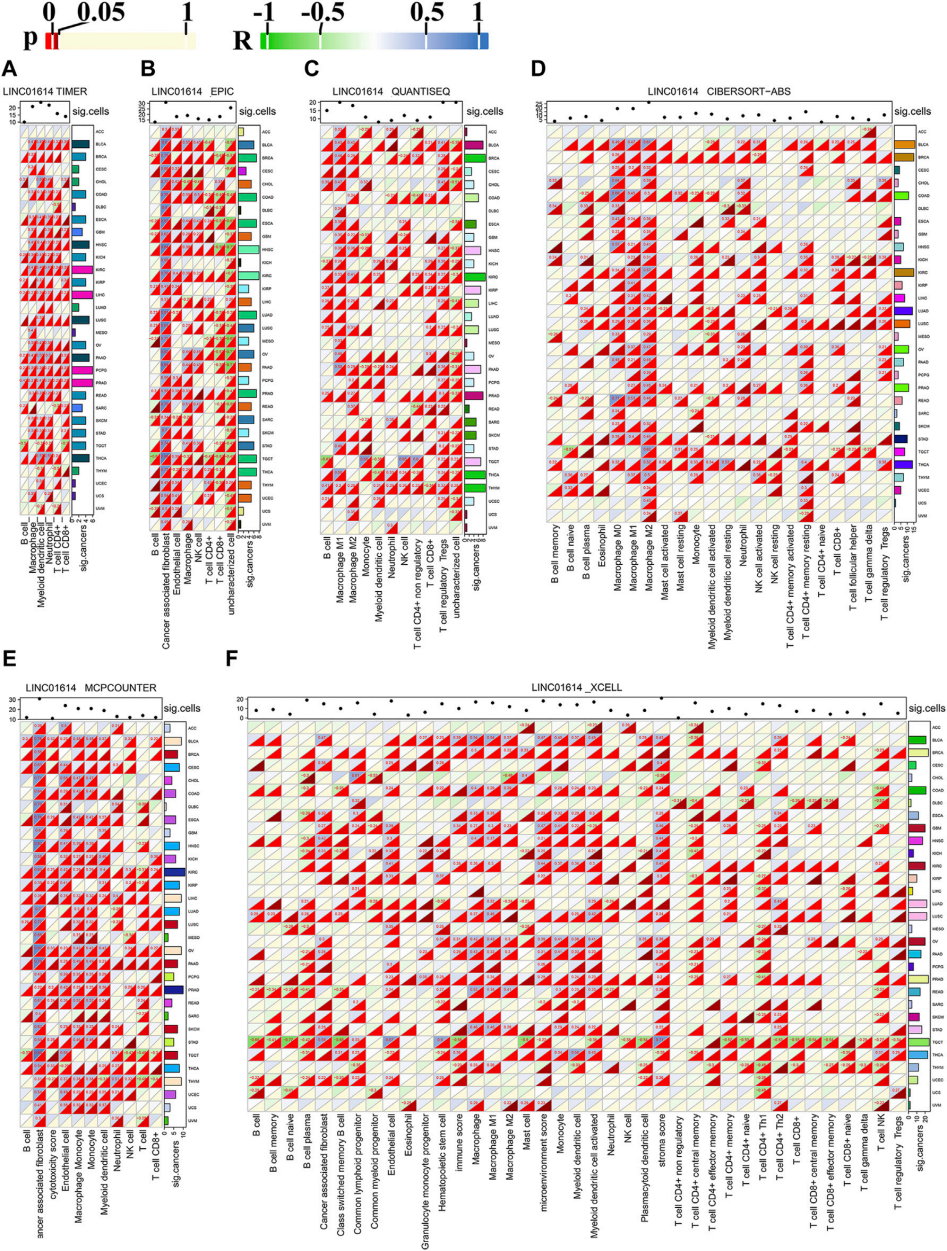
Pan-cancer correlation of LINC01614 expression and immune cell infiltration. The heatmap illustrates the Spearman correlation analysis results of LINC01614 expression and immune cell infiltration in pan-cancer, as determined by various methods: **(A)** TIMER, **(B)** EPIC, **(C)** QUANTISEQ, **(D)** CIBERSORT-ABS, **(E)** MCPCOUNTER, and **(F)** XCELL. Textual representation displays values with *p* < 0.05 and absolute r > 0.2. The sum of significant cancers or immune cell infiltrations is summarized on the left or the top of each heatmap.

The pan-cancer immune cell infiltration determined using the TIMER method showed that among 31 cancers, LINC01614 expression was correlated with six major immune cells: B cells, CD4^+^ T cells, CD8^+^ T cells, neutrophils, macrophages, and dendritic cells (Figure 5A). LINC01614 was positively correlated with aforementioned six major immune cells in PRAD and significantly correlated with dendritic cells in 26 cancers (Figure 5A). Significant positive correlations with macrophages in ESCA, HNSC, KIRC, LUSC, PRAD, and STAD were also verified by other five algorithms (Figures 5B–F). In BLCA, KIRC, OV, PAAD, pheochromocytoma and paraganglioma (PCPG), PRAD, and THCA, LINC01614 showed a significant correlation with dendritic cells, which was verified by QUANTISEQ, MCPCOUNTER, and XCELL methods (Figure 5C, E, F). The XCELL method illustrated that LINC01614 had a significant correlation with the microenvironment score in 22 cancers and with hematopoietic stem cell in 17 cancers (Figure 5F). EPIC and MCPCOUNTER and XCELL illustrated positive correlations between LINC01614 expression and cancer-associated fibroblasts in 19 cancers (Figure 5B, E, F). QUANTISEQ, CIBERSORT-ABS, MCPCOUNTER, and XCELL algorithms illustrated that LINC01614 was significantly correlated with neutrophils in COAD, BRCA, THCA, PAAD, THCA, and THYM (Figure 5C, D, E, F). All six methods also showed that LINC01614 was significantly correlated with B-cell infiltration in six cancer types, including a most positive significant correlation in BLCA, LUAD, and THCA, while a most negative correlation in BRCA, ESCA, and READ. For different CD4^+^ T cell types, LINC01614 showed significant correlations in 20 cancer types (Figures 5A–F). The most positive correlations were found in T cell CD4^+^ Th2 and T cell CD4^+^ memory cells. In contrast, most negative relations were found with T cell CD4^+^ Th1 and T cell CD4^+^ central memory cells (Figures 5A–F). These results elucidated that LINC01614 holds extensive influence on the infiltration of different immune cells in multiple cancers.

### 3.5 Relationship of LINC01614 expression with immunoregulatory genes

The significant correlation between LINC01614 and immune cell infiltration guided us to explore the influence of LINC01614 expression on the expression of immune-related genes. For chemokines, LINC01614 showed a significant correlation with more than half of the genes in most cancers, except in UCEC, MESO, ACC, UCS, uveal melanoma (UVM), PCPG, and CHOL. Seven chemokines (CCL18, CCL8, CXCL9, CXCL10, CXCL11, CCL26, and CCL7) showed positive relations in more than 25 cancer types (Supplementary Figure S3A). In LUSC, LUAD, ACC, and SKCM, LINC01614 showed negative relations with more than 10 chemokines of each cancer. CCL15, CCL14, CCL16, CCL23, and CCL27 showed negative relations in more than 10 cancers. With regard to immunostimulators, LINC01614 expression was positively correlated with 14/45 immunostimulators (ULBP1, TNFSF4, TNFRSF4, CD48, CXCR4, CD28, TNFSF18, CD86, TNFRSF9, CD80, IL2RA, NT5E, CD276, and ENTPD1) in more than 25 cancer types (Supplementary Figure S3E). A total of 10 cancers, namely, LIHC, PAAD, KIRP, PRAD, STAD, KIRC, OV, BLCA, SKCM, and KICH, showed positive relations with LINC01614 expression and more than 35 immunostimulators. Among these immunostimulators, TNFRSF25 and TNFRSF14 showed a negative coexpression in more than 10 cancers. In BRCA, COAD, ESCA, TGCT, LUSC, and LUAD, more than 10 immunostimulators showed negative relations with LINC01614 (Supplementary Figure S3E). As to immune inhibitors, KIRC, PAAD, SKCM, KIRP, STAD, BLCA, LGG, OV, and KICH cancers showed more than 20 positive relations with LINC01614. Nine inhibitors, namely, KDR, TGFB1, TGFBR1, CSF1R, HAVCR2, IDO1, IL10RB, CTLA4, and TIGIT, had a positive coexpression with LINC01614 in more than 25 cancers. In LUSC and LUAD, LINC01614 negatively correlated with more than 10 immune inhibitors (Supplementary Figure S3D). In KIRC, PAAD, PRAD, OV, STAD, BLCA, LIHC, and KICH, LINC01614 showed a positive coexpression with more than 15 receptors. CCR3, CCR8, CXCR4, CCR6, CX3CR1, CCR1, CCR2, CCR5, CXCR3, CXCR6, XCR1, and CCR4 positively coexpressed with LINC01614 in more than 20 cancers, while CXCR1 and CCR10 negatively coexpressed with LINC01614 in more than 10 cancers (Supplementary Figure S3B). A total 15 out of 21 MHC genes, namely, HLA-DMB, HLA-DQA2, HLA-DQB1, HLA-DMA, HLA-DOA, HLA-DQA1, HLA-DRB1, HLA-DRA, HLA-DPA1, HLA-DPB1, HLA-A, HLA-C, B2M, TAP1, and TAP2, positively coexpressed with LINC01614. HLA-F and HLA-E negatively coexpressed with LINC01614 in more than six cancers. Nine cancers, namely, LGG, LIHC, KIRC, PAAD, COAD, OV, STAD, BLCA, and KICH, showed negative relationships with more than 20 MHC genes, while LUSC showed 10 negative relations (Supplementary Figure S3C). This coexpression analysis verified an extensive influence of LINC01614 on immune-related genes. These may be a reason for its widespread impact on immune cell infiltration.

### 3.6 Relationship of LINC01614 expression with genomic instability parameters

**FIGURE 6:**
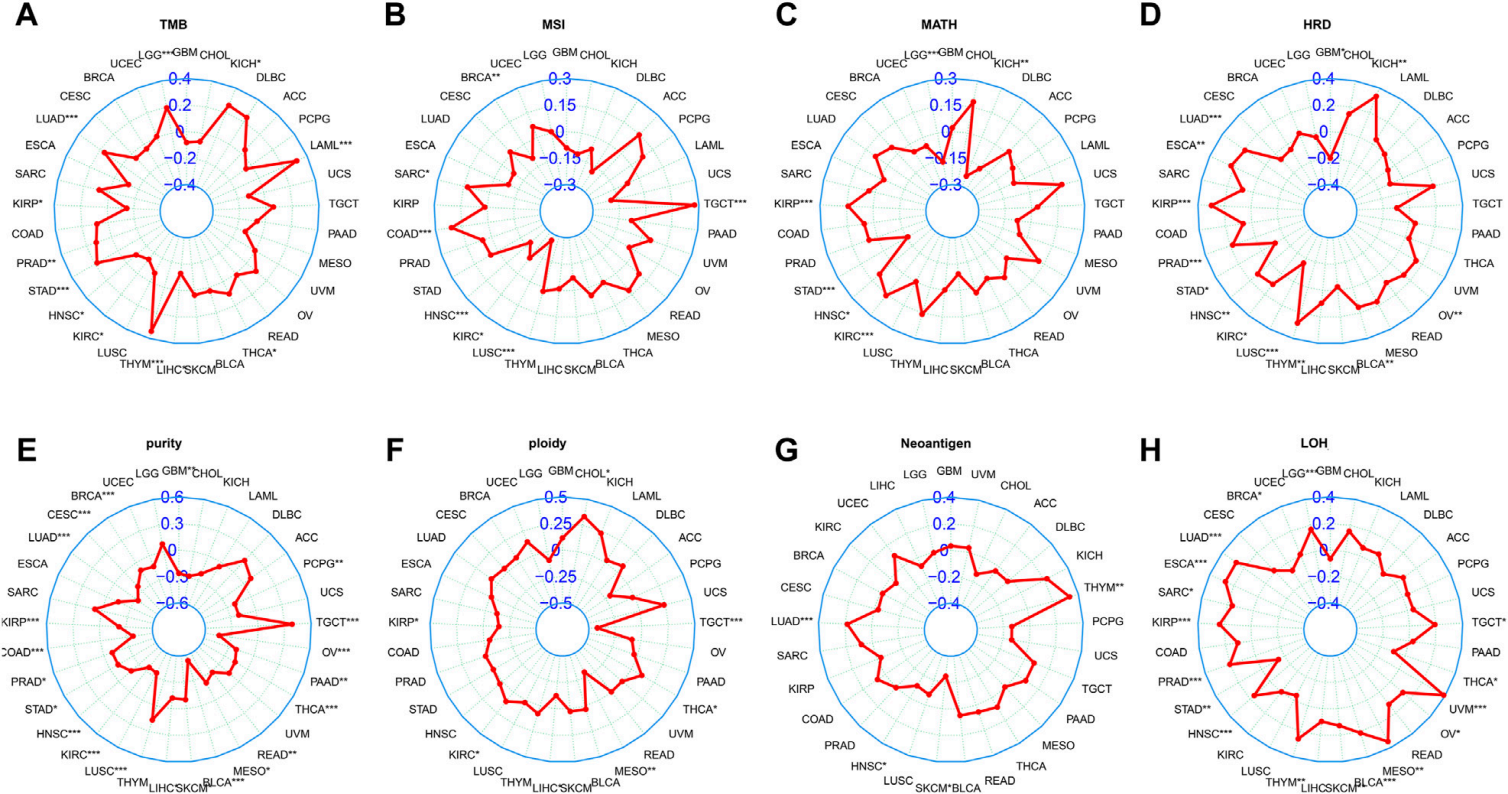
Correlation between LINC01614 and genomic instability parameters. Spearman correlation analysis was conducted to examine the relationship between the expression of LINC01614 expression and various genomic instability parameters, including **(A)** TMB, **(B)** MSI, **(C)** MATH, **(D)** HRD, **(E)** tumor purity, **(F)** ploidy, **(G)** neoantigens, and **(H)** LOH.

Currently, TMB, MSI, and MATH have been recognized as prognostic indicators of cancer immunotherapy (Chang et al., 2018). Our study showed that LINC01614 expression was positively correlated with TMB in LGG, LUAD, HNSC, KIRC, and LIHC and negatively correlated with TMB in KIRP, PRAD, STAD, THYM, THCA, acute myeloid leukemia (LAML), and KICH (Figure 6A). MSI of SARC, COAD, and TGCT patients showed positive correlations, while BRCA, HNSC, KIRC, and LUSC patients showed negative correlations with LINC01614 expression (Figure 6B). MATH for KIRP, HNSC, and KIRC patients showed positive correlations, while LGG, STAD, and KICH patients showed negative correlations with LINC01614 expression (Figure 6C). As for HRD, negative correlations were found in STAD and LUSC, while positive correlations were observed in LUAD, ESCA, KIRP, PRAD, HNSC, KIRC, THYM, BLCA, and KICH (Figure 6D). For tumor purity, 18 cancers showed negative correlations, while TGCT showed a positive correlation (Figure 6E). Ploidy of CHOL, KIRC, and THCA showed a positive correlation; by contrast, TGCT, MESO, and KIRP showed negative associations with LINC01614 expression ((Figure 6F). HNSC and SKCM showed negative associations of neoantigens and LINC01614 expression; on the contrary, LUAD and THYM presented a positive correlation (Figure 6G). For LOH, LINC01614 showed positive correlations in STAD, THCA, and BRCA but negative association in 12 cancers (LUAD, ESCA, SARC, KIRP, PRAD, HNSC, THYM, BLCA, MESO, UVM, OV, TGCT, and LGG) (Figure 6H). All of the above results strongly suggested that LINC01614 was a potential biomarker of genome stability in BRCA and LUAD. Collectively, these data implied that LINC01614 may influence antitumor immunity by regulating the composition and immune mechanism.

### 3.7 Relationship of LINC01614 expression with tumor stemness

**FIGURE 7:**
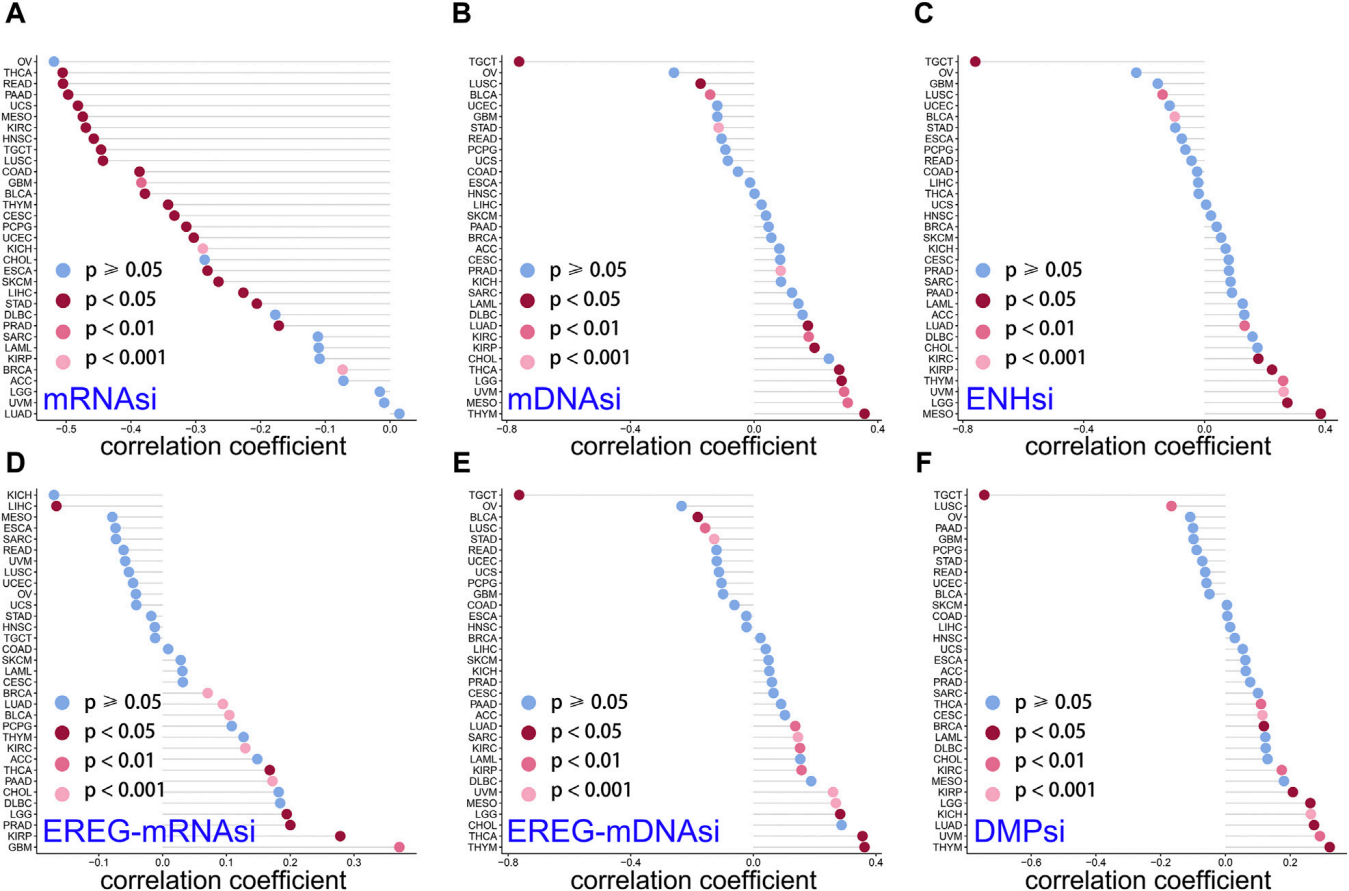
Correlation of LINC01614 and stemness. The Spearman correlation method was used to examine the relationship between LINC01614 expression and six stemness indicators, namely, **(A)** mRNAsi, **(B)** mDNAsi, **(C)** ENHsi, **(D)** EREG-mRNAsi, **(E)** EREG-mDNAsi, and **(F)** DMPsi.

Studies have revealed that tumor stemness could be used as a biomarker for predicting patient outcomes and therapy response (Najafi et al., 2019a). Cancer stem cells, due to their stem cell properties and self-renewal capabilities, play important roles in tumor cell progression and invasion (Reya et al., 2001). DMPsi; ENHsi and EREG-mRNAsi; and EREG-mDNAsi, mRNAsi, and mDNAsi are the six stemness indexes using a set of stemness features (Malta et al., 2018). Stemness correlation analysis showed that LINC01614 owned wide negative correlations with mRNAsi in 23 cancers (Figure 7A). For mDNAsi, LINC01614 expression showed positive correlations in LGG, LUAD, KIRP, PRAD, KIRP, PRAD, KIRC, THYM, THCA, MESO, and UVM, but negative correlations in LUSC, BLCA, STAD, and TGCT (Figure 7B). A positive correlation of LINC01614 and ENHsi was found in LGG, MESO, UVM, THYM, KIRP, KIRC, and LUAD; nevertheless, a negative correlation was found in LUSC and TGCT (Figure 7C). For EREG-mRNAsi, positive correlations existed in LGG, BRCA, LUAD, KIRP, PRAD, GBM, KIRC, BLCA, THCA, and PAAD but a negative correlation in LIHC (Figure 7D). Regarding EREG-mDNAsi, a positive association was shown in LGG, LUAD, SARC, KIRP, KIRC, THYM, THCA, MESO, and UVM. In contrast, in LGG, LUAD, SARC, KIRP, KIRC, THYM, THCA, MESO, and UVM, it showed a negative correlation with LINC01614 expression (Figure 7E). DMPsi was significantly positively correlated with 10 cancers, namely, LGG, BRCA, CESC, LUAD, KIRP, KIRC, THYM, THCA, UVM, and KICH, while negatively correlated with LUSC and TGCT with LINC01614 (Figure 7F). Although the mechanism of LINC01614 influence on cancer stemness remains not fully understood, our study indicated that LINC01614 could widely affect the stemness of different tumors.

### 3.8 Coexpression analysis of LINC01614 and RNA methylation-related genes

We explored the relationship among 42 RNA methylation-related genes associated with m6A, m5C, and mlA processes and found that LINC01614 expression was associated with most RNA-regulating genes in almost 26 cancers. LINC01614 was significantly associated with six M1A (TRMT6, TRMT10C, YTHDF2, YTHDF1, YTHDF3, and YTHDC1) (Supplementary Figure S4A), 19 M6A (RBM15, METTL14, WTAP, CBLL1, METTL14, ZC3H13, MEETTL3, RBM15B, FTO, FMR1, YTHDF3, YTHDC1, YTHDC2, YTHDF1, HNRNPA2B1, ELAVL1, YTHDF2, IGF2BP1, and IGF2BP3) (Supplementary Figure S4B), and eight M5C (NSUN3, TRDMT1, NSUN4, DNMT3A, NSUN2, DNMT3B, TET2, and ALYREF)-related genes in more than 20 tumors (Supplementary Figure S4C). For M1A, ALKBH3, TRMT61A, TRMT61B, YTHDF1, and YTHDC1 showed negative correlations in THCA (Supplementary Figure S4A). For M6A, METTL3 and YTHDC2 were negatively correlated with THCA, LUSC, LUAD, SKCM, and TGCT (Supplementary Figure S4B). As for M5C, NSUN7, TRDMT1, NOP2, NSUN2, DNMT3B, NSUN5, NSUN5, NSUN6, and TET2 were negatively correlated with THCA (Supplementary Figure S4C). PAAD was positively correlated with more than two-third of methylation-related genes of m6A, m5C, and mlA (Supplementary Figure S4A–C). The correlation analysis showed that LGG, LIHC, PAAD, BRCA, LUSC, SKCM, COAD, KIRC, LUAD, STAD, HNSC, and TGCT had a universal strong correlation between LINC01614 and methylation-related genes. These results indicated that LINC01614 might be involved in RNA modification.

### 3.9 GSEA of LINC01614 hallmark pathways

**FIGURE 8:**
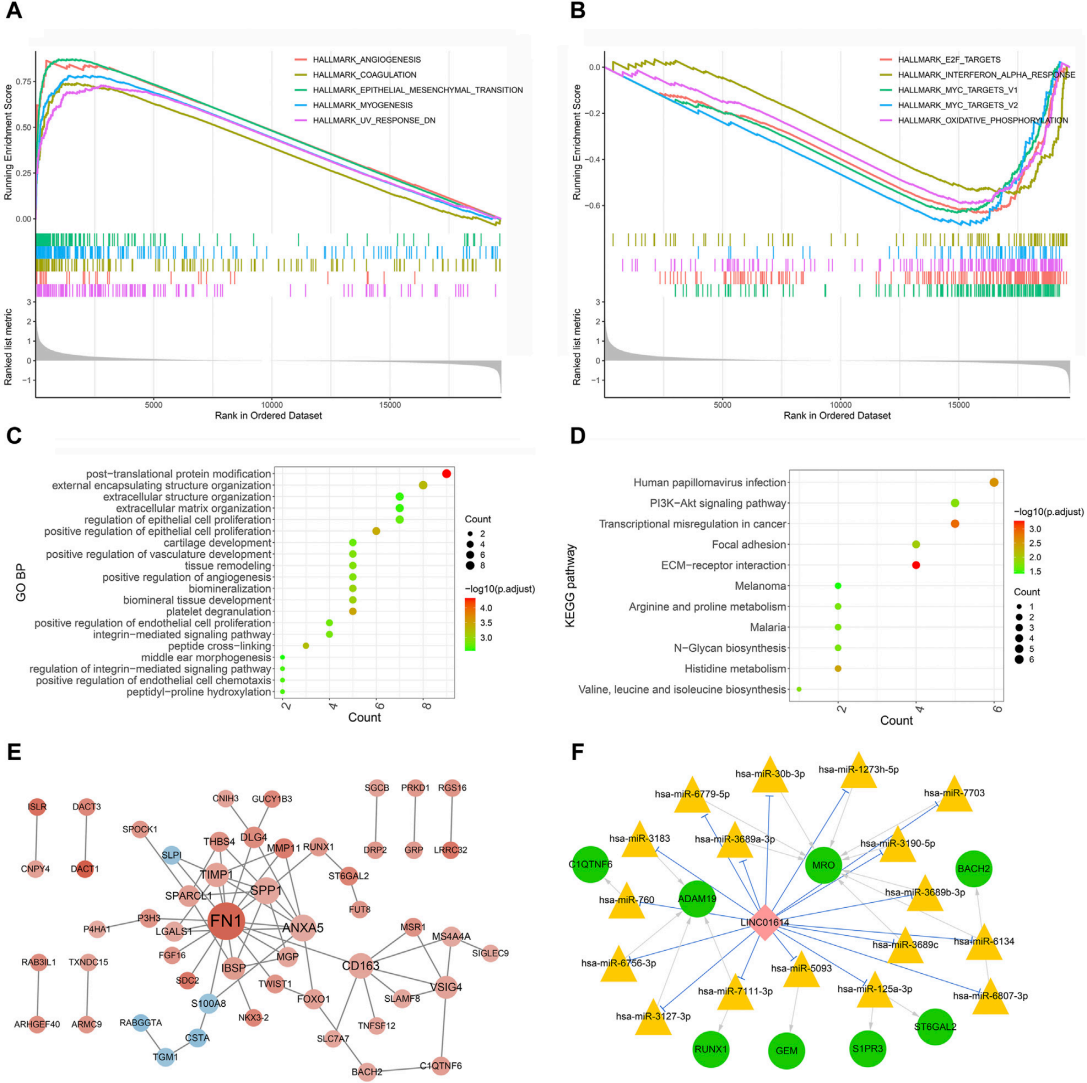
Functional enrichment and coexpression network analysis of LINC01614 in HNSC. The GSEA results of the top five upregulated hallmark pathways **(A)** and the top five downregulated hallmark pathways **(B)** associated with LINC01614 expression. The top 20 GO functional items **(C)** and the 11 significant KEGG functional items **(D)** enriched for the LINC01614 coexpressed metastasis-associated genes. **(E)** PPI network of LINC01614 coexpressed genes, with red indicating a positive correlation and blue indicating a negative correlation. The intensity of the color represented the magnitude of the absolute correlation coefficient, while the size of the node indicated the level of connectivity **(F)** The ceRNA networks of LINC01614 were illustrated, with green circles representing mRNA, pink diamond representing LncRNA, yellow triangles representing miRNA, blue linkage representing competitive binding of lncRNA to miRNA, and gray linkage representing miRNA-regulated mRNA.

GSEA of hallmark pathways identified a total of 27 enriched pathways, including 17 upregulated and 10 downregulated pathways (Supplementary Table S1). The top five upregulated and downregulated pathways were chosen for visualization (Figure 8A, B). Among the top enriched functions, hallmark pathways such as epithelial–mesenchymal transition, angiogenesis, myogenesis, coagulation, and UV response DN were positively associated with LINC01614 expression (Figure 8A). Contrastingly, hallmark pathways such as oxidative phosphorylation, interferon alpha response, MYC_targets V1, MYC_targets V2, and E2F target pathways were downregulated. These results suggest that the poor prognosis role of LINC01614 may also be related to its influence on different tumor hallmark pathways (Figure 8B).

### 3.10 Functional enrichment and PPI network construction of LINC01614 metastasis-associated coexpressed genes

The Kruskal–Wallis test identified 2,708 mRNAs that are related to pathologic_N transfer. Through coexpression analysis of LINC01614 and these pathologic N transfer-related genes by Pearson correlation analysis (absolute R > 0.4, *p* < 0.05), a total of 101 LINC01614 coexpressed genes were screened (Supplementary Table S2). GO biological process (BP) and KEGG pathway enrichment analyses, based on the aforementioned LINC01614 coexpressed genes, were significantly enriched in a total of 166 GO biological pathways and 11 KEGG pathways (Supplementary Table S3). The top 20 significant GO pathways and significant KEGG pathways are shown in Figure 8C, D, respectively. The cross-linking, positive regulation of epithelial cell proliferation, platelet degranulation, and other pathways were significantly enriched. The KEGG pathway analysis showed that the PI3K-Akt signaling pathway, extracellular matrix (ECM) receptor, histidine metabolism, and cell adhesion pathways were significantly enriched. Furthermore, among these 101 LINC01614 coexpressed metastasis-associated genes, a total of 66 interaction pairs consisting of 53 genes were constructed in the PPI network (Figure 8C; Supplementary Table S4). As showed by the PPI network, LINC01614 was positively associated with 48 genes and negatively with five genes. FN1, SPP1, ANXA5, CD163, TIMP1, IBSP, and VSIG4 all had more than five interactions with each other, directly or indirectly (Figure 8C).

### 3.11 ceRNA network construction

From the miRNA screening process, a total of 193 miRNAs (Supplementary Table S5) were predicted as targets of LINC01614 coexpressed genes and 118 miRNAs (Supplementary Table S6) were predicted to directly target LINC01614. Furthermore, LINC01614–miRNA–mRNA correlation pairs were constructed by the significant positive coexpression of mRNAs and LINC01614, which were targeted by the same miRNAs. In total, 20 LINC01614–miRNA–mRNA relationships were then obtained. Within these ceRNA networks, the genes that had top two interactions were the *MRO* gene (hsa-miR-6779-5p, hsa-miR-30b-3p, hsa-miR-1273h-5p, hsa-miR-7703, hsa-miR-3190-5p, hsa-miR-3689b-3p, hsa-miR-6134, hsa-miR-3689c, and hsa-miR-3689a-3p) and the *ADAM19* gene (hsa-miR-3183, hsa-miR-760, hsa-miR-6756-3p, hsa-miR-3127-3p, and hsa-miR-7111-3P) (Figure 8F).

### 3.12 LINC01614 is associated with drug sensitivity in pan-cancer

We explored potential compounds capable of targeting pan-cancer based on the IC_50_ available in the GDSC-V2 database for each TCGA sample. Excitingly, 17 chemotherapeutic compounds were selected for which the estimated IC_50_ values were significantly influenced by LINC01614 in HNSC. Among these significantly influenced drugs, we found that LINC01614 expression was sensitive to Wnt-C59, dihydrorotenone, and axitinib (Supplementary Figure S5) but resistant to Nu7441. In addition, LINC01614 was positively correlated with Wnt-C59, ribociclib, OSI-027, leflunomide, dihydrorotenone, dactinomycin, axitinib, and AGI-5198. It was negatively correlated with Sb505124, RVX-208, RO-3306, PD173074, NU-7441, JQ1, BI-2536, and AZD8055 in HNSC. The total cancer samples also showed a significant influence on OSI-027 and BI-2536. These findings further suggested that the expression level of LINC01614 in pan-cancer patients might be related to the effect of chemotherapy.

### 3.13 The biofunction of LINC01614 in HNSC illustrated by *in vitro* assays

**FIGURE 9:**
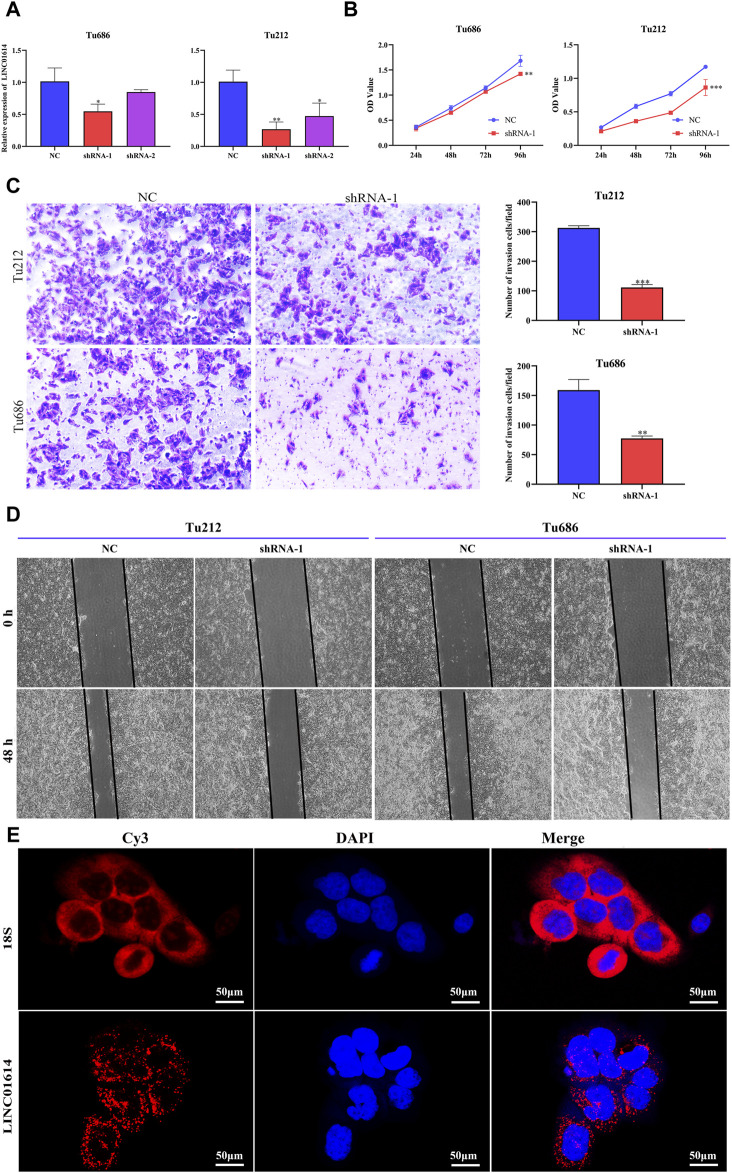
The biological function of LINC01614 in HNSC was investigated *in vitro*. **(A)** The silencing efficiency of siRNA targeting LINC01614 was analyzed by qPCR 48 h after transfection in TU212 or TU686 cells. **(B)** The effect of LINC01614 knockdown on cell proliferation was tested using CCK-8 assay. **(C)** Transwell assays were performed to test the migration and invasion activity of LINC01614-altered cells. All data are presented as the mean ± standard deviation of three experiments. Statistical significance was indicated as* *p* < 0.05, **p < 0.01, ***p < 0.001, and ****p < 0.0001. **(D)** Wound healing assays indicated that knockdown of LINC01614 could restrain the migration of TU212 or TU686 cells. **(E)** RNA FISH was conducted to visualize LINC01614 in tumor cells.

To explore the biofunction of LINC01614 in HNSC, its expression was significantly knocked down in Tu212 and Tu686 cells transfected with shRNA-LINC01614 (Figure 9A). The CCK-8 assay showed that LINC01614 knockdown hindered HNSC cell proliferation compared with the negative control (NC) cells (Figure 9B). Furthermore, wound healing and transwell assay results further confirmed that the migration and invasion ability of HNSC cells were weakened in LINC01614 knockdown cells (Figure 9C, D). As shown in Figure 9E, the FISH results showed that LINC01614 was mainly distributed in the cytoplasm of HNSC cells. Our results suggested that altered LINC01614 expression might affect cell growth and migration in HNSC cells.

## 4 Discussion

Studies have indicated that abnormalities in certain lncRNAs are closely related to tumor progression by influencing different biological functions, such as neoplastic proliferation, tumor differentiation, invasion, metastasis, angiogenesis, resistance to cell death, tumor-promoting inflammation, cancer stemness, or genome instability (Bhan et al., 2017; Yan and Bu, 2021). Accumulating evidence indicated that LINC01614 was abnormally expressed in diverse malignant tumor tissues, including osteosarcoma (Cai et al., 2021), gastric cancer (Wu et al., 2021), glioma (Wang et al., 2020b), breast cancer (Wang et al., 2019a), and lung cancer (Sun and Ling, 2019), and was closely correlated with clinical pathology indicators and could predict prognosis (Wang et al., 2020a; Dong et al., 2021). Knockdown of LINC01614 inhibited cell growth, migration, and invasion of THCA and esophageal squamous cell carcinoma (Wei et al., 2023). In BRCA, LINC01614 as a hazard prognosis factor was associated with the HR+/HER2 + molecular subtype (Li et al., 2023). In osteosarcoma, LINC01614 as a ceRNA promoted tumor progression through the miR-520a-3p/SNX3 axis (Cai et al., 2021). In glioma, the upregulation of LINC01614 by SP1 promoted cancer progression by regulating the miR-383/ADAM12 axis (Wang et al., 2020b). Although studies still need further investigation, these results suggested that LINC01614 could function as a cytoplasmic lncRNA in various cancers, and its upregulation could cause poor prognosis.

In this study, we found that 23 out of 30 cancers showed a significant abnormal expression of LINC01614. This irregular expression also predicted a significant diagnostic value in distinguishing tumor and normal samples of nine cancers with AUC values of the ROC curve above 0.85. Tumor stage correlation analysis showed that the most significant differences of LINC01614 occurred in advanced pathological stages in more than 11 cancers. The correlation analysis of molecular subtypes revealed significant differences in LINC01614 expression among seven distinct tumor subtypes. Prognosis analysis showed that the LINC01614 expression level was associated with prognosis of 26 cancer types across one to five different survival outcomes. Among these, it is only in DLBC that a benign prognosis role for OS, PFS, and PFI was observed. An unfavorable influence on all five different survival endpoints was found in seven cancers. These results suggested that LINC01614 might serve as an important prognostic and diagnostic biomarker in most cancers.

The tumor microenvironment (TME) refers to the internal and external environments of tumor cells that are closely related to the tumor occurrence, growth, and metastasis. The TME alteration could influence tumor cell growth and spread. Additionally, immune cells, immune mediators, and cell receptors in the TME play a key role in the immune response (Hinshaw and Shevde, 2019). Unlike the conventional view of immune cells being involved in antitumor immunity, immune infiltration cells in the TME can reflect a tactic that tumor cells avoid being killed (Gajewski et al., 2013). The infiltration of different immune cells including activated T cells, dendritic cells, neutrophils, and macrophages cells is a critical factor in antitumor immunity and has a direct impact on tumor development, tumor cell proliferation, and immunosuppression (Veglia and Gabrilovich, 2017; Najafi et al., 2019b; Hinshaw and Shevde, 2019; Mollinedo, 2019). LncRNAs that affect tumor immune microenvironments and as potential tumor immunotherapy targets have become a new research focus. Our analysis showed that LINC01614 expression was correlated with the microenvironment score, hematopoietic stem cells, B cells, CD4^+^ T cells, CD8^+^ T cells, neutrophils, macrophages, and dendritic cells in 31 cancers, suggesting that LINC01614 influences tumor immune cell infiltration. Moreover, a significant correlation between LINC01614 and the tumor microenvironment had also been reported in papillary thyroid carcinoma, previously (Dong et al., 2021).

Loss of HLA can cause inefficient ability of presenting neoantigens to promote immune evasion (Aptsiauri and Garrido, 2022). Expressions of HLA-A, HLA-B, HLA-C, and HLA-G were associated with prognosis of different cancers (Noblejas-López et al., 2019; Michelakos et al., 2022; Xu et al., 2022). HLA-DRA and HLA-DMA expressions were synergistically related to immune checkpoint blockade efficacy, T-cell rejection, and high risk of recurrence for estrogen receptor (ER)-negative cells (Liu and Hofman, 2022). Our results unraveled a strong positive link between LINC01614 and MHC genes, such as HLA-DMB, HLA-DQA2, HLA-DQB1, HLA-DMA, HLA-DOA, HLA-DQA1, HLA-DRB1, HLA-DRA, HLA-DPA1, HLA-DPB1, HLA-A, and HLA-C, in multiple cancers. This significant pan-cancer coexpression of LINC01614 with MHC classes suggests its potential functional influence on cancer immunotherapy.

Immunity checkpoint therapies use antibodies to destroy cells' immunoregulatory checkpoints and release pre-existing antitumor immune reactions. Our study showed that LINC01614 was significantly coexpressed with more than half of the chemokines or more than 14 immunostimulators in most cancers. Among these coexpressed immunostimulators, the role of highly expressed CD276 (B7-H3) as an immune checkpoint target in cancer cells is inhibition of T-cell function and CD8^+^ T-cell infiltration (Miyamoto et al., 2022). In squamous cell carcinoma, CD276 promoted evasion of immune surveillance by carcinoma stem cells and inhibited their clearance by CD8^+^ T cells (Wang et al., 2021a). Combined with the previous studies, our results suggest that LINC01614 might mediate CD276-induced stemness maintenance and CD8^+^ T-cell suppression.

The immune response is often correlated with somatic variation. Genome instability or mutation evaluation indicators such as MSI, TMB, and neoantigens play important roles in tumorigenesis. TMB and MSI can lead to neoantigen formation and anti-tumor immune response activation (Liberzon et al., 2015; Ganesh et al., 2019). Tumors with high levels of TMB and MSI respond to better immunotherapy. Our analysis illuminated that LINC01614 expression was significantly related to MSI, TMB, or neoantigen in 18 cancers. Among these cancers, HNSC showed a negative correlation of LINC01614 expression with MSI, TMB, and neoantigen. LOH is a chromosomal event that can cause loss of entire genes and nearby chromosomal regions. LOH, HRD, and intratumoral heterogeneity are associated with immune infiltration (Nguyen et al., 2020). We found in a total of eight cancers that LINC01614 displayed a significant correlation with both HRD and LOH, including seven positive correlations (LUAD, ESCA, KIRP, PRAD, HNSC, THYM, and BLCA) and one negative correlation (STAD). For ploidy and purity, KIRC and THCA patients showed positive correlations, while TGCT showed a negative correlation. These significant influences on TMB, MSI, MATH, HRD, purity, ploidy, neoantigen, and LOH suggested that LINC01614 might serve as an important biomarker for immunotherapy prognosis evaluation, especially in HNSC patients.

Cancer stemness refers to the progressive loss of cancer cell-differentiated phenotype and the acquisition of progenitor-like and stem cell-like features. Cancer stemness is associated with cancer metastasis, drug resistance, recurrence, and poor prognosis (Qin et al., 2020; Zhao et al., 2020). Studies have indicated that tumor stemness influences cancer prognosis of different cancers. For example, mDNAsi expression was significantly correlated with OS in UVM (Wan et al., 2023). In adrenocortical carcinoma, high mRNAsi was associated with poor OS and increased metastatic potential (Shi et al., 2021). Our analysis indicated that LINC01614 was significantly correlated with different stemness indicators in various cancers. In LGG, LUAD, KIRP, and KIRC, LINC01614 showed a significant positive correlation with mDNAsi, ENHsi, EREG-mRNAsi, EREG-mDNAsi, and DMPsi. Only in LUSC and TGCT, LINC01614 expression showed a negative correlation with DMPsi, ENHsi, mDNAsi, and mRNAs. These results revealed that LINC01614 might promote tumor stemness in different cancers.

A previous review showed that tumor stemness can be influenced by m6A. For example, m6A writer METTL3 could stimulate stemness of GBM, bladder cancer, cSCC, and osteosarcoma (Wang et al., 2019b; Cheng et al., 2019; Gao et al., 2020; Zhang et al., 2021). The m^6^A modification also showed an influence on tumorigenesis, progression, migration, and invasion (Chen et al., 2019). The m6A methylation plays an essential role in the progression of bladder and gastric cancers (Wang JZ. et al., 2021; Li et al., 2021). Elevated expression of m6A-modified CENPK in cervical cancer was associated with cancer recurrence and independently predicted poor prognosis. The m5C regulator NSUN2 promoted cervical cancer cell migration and invasion by inducing m5C methylation of keratin 13 (KRT13) transcripts (Wang L. et al., 2022). High expression of m1A-related regulators (m1A writer TRMT6, TRMT61A, and eraser ALKBH3) was associated with poor prognosis in several cancers, including hepatocellular carcinoma, colon cancer, glioma, breast cancer, and ovarian cancer (Woo and Chambers, 2019; Shi et al., 2020; Wang B. et al., 2021; Gao et al., 2021).

In this study, LINC01614 expression was positively correlated with regulatory genes of RNA methylation for several cancers. Among these strong RNA methylation-related genes and LINC01614 coexpressed cancers, PAAD was positively correlated with more than two-thirds of methylation-related genes of m6A, m5C, and m1A, suggesting that coexpression of methylation-related genes with LINC01614 might be one reason for its poor prognosis influence on PAAD. In summary, our findings showed that LINC01614 has a wider coexpression with RNA methylation-related genes.

According to the drug sensitivity analysis, we found that LINC01614 expression showed a positive response to chemotherapy-responsive drugs (Wnt-C59, dihydrorotenone, and axitinib), whereas a negative response to Nu7441 in HNSC, KICH, KIRP, SKCM, and TGCT patients was observed. LINC01614 was positively correlated with Wnt-C59, ribociclib, OSI-027, leflunomide, dihydrorotenone, dactinomycin, axitinib, and AGI-5198 and negatively correlated with Sb505124, RVX-208, RO-3306, PD173074, NU-7441, JQ1, BI-2536, and AZD8055 in HNSC. Axitinib is a small-molecule anticancer drug that inhibits angiogenesis. Axitinib is effective in treating some tumors, including HNSC, KIRC, KIRP, THCA, and SKCM (Zhang et al., 2015; Jiang et al., 2023). These findings suggested that LINC01614 expression might help guide clinical drug choice and the prognosis of patients with particular cancers.

Since LINC01614 has a potential cancer-promoting effect in various cancers, including HNSC, and the biological function and oncogenic role of LINC01614 in HNSC were then tested *in vivo* in HNSC cell lines. LINC01614 knockdown remarkably impaired the proliferation, migration, and invasion abilities of both Tu212 and Tu686 cells. FISH staining further confirmed that LINC01614 RNA is predominantly localized to the cytoplasm. GSEA results showed that LINC01614 expression was mainly highly enriched in epithelial–mesenchymal transition, angiogenesis, coagulation, myogenesis, and other pathways. These pathways are closely related to tumorigenesis and progression and are consistent with the function of LINC01614 tested *in vitro*. These pathways are implicated in its poor prognosis for HNSC patients.

In addition, we focused on the correlation between LINC01614 and lymph node metastasis genes and obtained 101 coexpressed genes related to LINC01614 metastasis by correlation analysis. Functional enrichment analysis showed that many cancers associated GO terms or KEGG pathways were significantly enriched. This was supported by a previous study that LINC01614 also played a crucial role in the PI3K-Akt signaling pathway, ECM receptor interaction, and promoted tumor progression (Yan et al., 2021). The engagement of these pathways is important in accelerated tumor proliferation and metastasis. The influence on prognosis and diagnosis, along with tumor-associated pathway enrichment, suggests that LINC01614 might be an indicator for the treatment of HNSC.

LncRNAs act as ceRNAs to absorb miRNAs through sequence complementation and subsequently impact the functional role of miRNAs. LINC01614 located in the cytoplasm can function as a cytoplasmic lncRNA, and its upregulation can cause poor prognosis in various cancers.

## 5 Conclusion

Our pan-cancer analysis revealed that LINC01614 expression was associated with diagnosis, prognosis, immune cell infiltration, immune checkpoint genes, MATH, TMB, MSI, and tumor stemness. LINC01614 might influence HNSC progression through regulating EMT, angiogenesis, immune infiltration, and TME. Therefore, LINC01614 has the potential to serve as a biomarker for evaluating the prognosis or a target for the treatment of HNSC patients.

## Figures and Tables

**Table:** 

**ACC**	adrenocortical carcinoma
**BLCA**	bladder urothelial carcinoma
**BRCA**	breast invasive carcinoma
**CESC**	cervical and endocervical cancer
**CHOL**	cholangiocarcinoma
**COAD**	colon adenocarcinoma
**DLBC**	Lymphoid Neoplasm Diffuse Large B-cell Lymphoma
**ESCA**	esophageal carcinoma
**ESCC**	esophageal squamous cell carcinoma
**GBM**	glioblastoma multiforme
**HNSC**	head and neck squamous cell carcinoma
**KICH**	kidney chromophobe
**KIRC**	kidney renal clear cell carcinoma
**KIRP**	kidney renal papillary cell carcinoma
**LAML**	acute myeloid leukemia
**LGG**	brain lower-grade glioma
**LIHC**	liver hepatocellular carcinoma
**LUAD**	lung adenocarcinoma
**LUSC**	lung squamous cell carcinoma
**OV**	ovarian serous cystadenocarcinoma
**PAAD**	pancreatic adenocarcinoma
**PCPG**	pheochromocytoma and paraganglioma
**PRAD**	prostate adenocarcinoma
**READ**	rectum adenocarcinoma
**SARC**	sarcoma
**SKCM**	skin cutaneous melanoma
**STAD**	stomach adenocarcinoma
**TGCT**	testicular germ cell tumor
**THCA**	thyroid carcinoma
**THYM**	thymoma
**UCEC**	uterine corpus endometrial carcinoma
**UCS**	uterine carcinosarcoma
**UVM**	uveal melanoma

## Data Availability

The datasets presented in this study can be found in online repositories. The names of the repository/repositories and accession number(s) can be found in the article/Supplementary Material.

## References

[B1] Aptsiauri N. Garrido F. (2022). The challenges of hla class I loss in cancer immunotherapy: facts and hopes. Clin. Cancer Res. 28 (23), 5021–5029. 10.1158/1078-0432.CCR-21-3501 35861868

[B2] Bagchi S. Yuan R. Engleman E. G. (2021). Immune checkpoint inhibitors for the treatment of cancer: clinical impact and mechanisms of response and resistance. Annu. Rev. Pathol. 16, 223–249. 10.1146/annurev-pathol-042020-042741 33197221

[B3] Bhan A. Soleimani M. Mandal S. S. (2017). Long noncoding rna and cancer: a new paradigm. Cancer Res. 77 (15), 3965–3981. Epub 2017/07/14. 10.1158/0008-5472.CAN-16-2634 28701486 PMC8330958

[B4] Bonneville R. Krook M. A. Kautto E. A. Miya J. Wing M. R. Chen H.-Z. (2017). Landscape of microsatellite instability across 39 cancer types. JCO Precis. Oncol. 2017, 1–15. 10.1200/PO.17.00073 PMC5972025 29850653

[B5] Cai Q. Zhao X. Wang Y. Li S. Wang J. Xin Z. (2021). Linc01614 promotes osteosarcoma progression via mir-520a-3p/snx3 Axis. Cell Signal 83, 109985. Epub 2021/03/24. 10.1016/j.cellsig.2021.109985 33753211

[B6] Cai Y. Wu S. Jia Y. Pan X. Li C. (2022). Potential key markers for predicting the prognosis of gastric adenocarcinoma based on the expression of ferroptosis-related lncrna. J. Immunol. Res. 2022, 1249290. 10.1155/2022/1249290 35528617 PMC9076347

[B7] Chang L. Chang M. Chang H. M. Chang F. (2018). Microsatellite instability: a predictive biomarker for cancer immunotherapy. Appl. Immunohistochem. Mol. Morphol. 26 (2), e15–e21. 10.1097/PAI.0000000000000575 28877075

[B8] Chen X.-Y. Zhang J. Zhu J.-S. (2019). The role of M6a rna methylation in human cancer. Mol. Cancer 18 (1), 103. 10.1186/s12943-019-1033-z 31142332 PMC6540575

[B9] Chen Y. Cheng W. Y. Shi H. Huang S. Chen H. Liu D. (2021). Classifying gastric cancer using flora reveals clinically relevant molecular subtypes and highlights Linc01614 as a biomarker for patient prognosis. Oncogene 40 (16), 2898–2909. Epub 2021/03/21. 10.1038/s41388-021-01743-3 33742127 PMC8062268

[B10] Cheng M. Sheng L. Gao Q. Xiong Q. Zhang H. Wu M. (2019). The M(6)a methyltransferase Mettl3 promotes bladder cancer progression via aff4/nf-κb/myc signaling network. Oncogene 38 (19), 3667–3680. Epub 2019/01/20. 10.1038/s41388-019-0683-z 30659266

[B11] de Santiago P. R. Blanco A. Morales F. Marcelain K. Harismendy O. Sjöberg Herrera M. (2021). Immune-related incrna Linc00944 responds to variations in Adar1 levels and it is associated with breast cancer prognosis. Life Sci. 268, 118956. Epub 2021/01/01. 10.1016/j.lfs.2020.118956 33383047

[B12] Dong X. Jin C. Chen D. Chen Y. Ye Z. Q. Zhang X. (2021). Genomic instability-related lncrna signature predicts the prognosis and highlights Linc01614 is a tumor microenvironment-related oncogenic lncrna of papillary thyroid carcinoma. Front. Oncol. 11, 737867. Epub 2021/10/05. 10.3389/fonc.2021.737867 34604079 PMC8481916

[B13] Gajewski T. F. Schreiber H. Fu Y.-X. (2013). Innate and adaptive immune cells in the tumor microenvironment. Nat. Immunol. 14 (10), 1014–1022. 10.1038/ni.2703 24048123 PMC4118725

[B14] Ganesh K. Stadler Z. K. Cercek A. Mendelsohn R. B. Shia J. Segal N. H. (2019). Immunotherapy in colorectal cancer: rationale, challenges and potential. Nat. Rev. Gastroenterology hepatology 16 (6), 361–375. Epub 2019/03/20. 10.1038/s41575-019-0126-x 30886395 PMC7295073

[B15] Gao Q. Zheng J. Ni Z. Sun P. Yang C. Cheng M. (2020). The M(6)a methylation-regulated Aff4 promotes self-renewal of bladder cancer stem cells. Stem cells Int. 2020, 8849218. Epub 2020/07/18. 10.1155/2020/8849218 32676121 PMC7352121

[B16] Gao Y. Wang H. Li H. Ye X. Xia Y. Yuan S. (2021). Integrated analyses of M(1)a regulator-mediated modification patterns in tumor microenvironment-infiltrating immune cells in colon cancer. Oncoimmunology 10 (1), 1936758. Epub 2021/07/06. 10.1080/2162402x.2021.1936758 34221700 PMC8224220

[B17] Han X. Wang M. Zhao Y. L. Yang Y. Yang Y. G. (2021). Rna methylations in human cancers. Semin. Cancer Biol. 75, 97–115. Epub 2020/11/22. 10.1016/j.semcancer.2020.11.007 33220459

[B18] Hinshaw D. C. Shevde L. A. (2019). The tumor microenvironment innately modulates cancer progression. Cancer Res. 79 (18), 4557–4566. 10.1158/0008-5472.CAN-18-3962 31350295 PMC6744958

[B19] Huang T.-X. Fu L. (2019). The immune landscape of esophageal cancer. Cancer Commun. (Lond) 39 (1), 79. 10.1186/s40880-019-0427-z 31771653 PMC6878621

[B20] Huarte M. (2015). The emerging role of lncrnas in cancer. Nat. Med. 21 (11), 1253–1261. 10.1038/nm.3981 26540387

[B21] Jiang H. Liao J. Wang L. Jin C. Mo J. Xiang S. (2023). The multikinase inhibitor Axitinib in the treatment of advanced hepatocellular carcinoma: the current clinical applications and the molecular mechanisms. Front. Immunol. 14, 1163967. Epub 2023/06/16. 10.3389/fimmu.2023.1163967 37325670 PMC10264605

[B22] Li T. Fu J. Zeng Z. Cohen D. Li J. Chen Q. (2020). Timer2.0 for analysis of tumor-infiltrating immune cells. Nucleic Acids Res. 48 (W1), W509–W14. 10.1093/nar/gkaa407 32442275 PMC7319575

[B23] Li T. Wang T. Jing J. Sun L. (2021). Expression pattern and clinical value of key M6a rna modification regulators in abdominal aortic aneurysm. J. Inflamm. Res. 14, 4245–4258. Epub 2021/09/14. 10.2147/jir.S327152 34511965 PMC8412829

[B24] Li W. Cheng Y. Cheng J. Yao J. Song M. Yan M. (2023). Long noncoding rna Linc01614 is a diagnostic and prognostic marker for breast cancer. Discov. Med. 35 (174), 19–27. Epub 2023/04/07. 10.24976/Discov.Med.202335174.3 37024438

[B25] Li X. Song Y. (2020). Proteolysis-targeting chimera (protac) for targeted protein degradation and cancer therapy. J. Hematol. Oncol. 13 (1), 50. 10.1186/s13045-020-00885-3 32404196 PMC7218526

[B26] Liberzon A. Birger C. Thorvaldsdóttir H. Ghandi M. Mesirov J. P. Tamayo P. (2015). The molecular signatures database (msigdb) hallmark gene set collection. Cell Syst. 1 (6), 417–425. 10.1016/j.cels.2015.12.004 26771021 PMC4707969

[B27] Liu A.-N. Qu H.-J. Yu C.-Y. Sun P. (2018a). Knockdown of Linc01614 inhibits lung adenocarcinoma cell progression by up-regulating mir-217 and down-regulating Foxp1. J. Cell Mol. Med. 22 (9), 4034–4044. 10.1111/jcmm.13483 29934982 PMC6111824

[B28] Liu D. Hofman P. (2022). Expression of Notch1, Notch4, hla-dma and hla-dra is synergistically associated with T cell exclusion, immune checkpoint blockade efficacy and recurrence risk in Er-negative breast cancer. Cell Oncol. (Dordr) 45 (3), 463–477. 10.1007/s13402-022-00677-6 35543859 PMC12978109

[B29] Liu J. Lichtenberg T. Hoadley K. A. Poisson L. M. Lazar A. J. Cherniack A. D. (2018b). An integrated tcga pan-cancer clinical data resource to drive high-quality survival outcome analytics. Cell 173 (2), 400–416.e11. Epub 2018/04/07. 10.1016/j.cell.2018.02.052 29625055 PMC6066282

[B30] Liu T. Han C. Fang P. Ma Z. Wang X. Chen H. (2022). Cancer-associated fibroblast-specific lncrna Linc01614 enhances glutamine uptake in lung adenocarcinoma. J. Hematol. Oncol. 15 (1), 141. Epub 2022/10/09. 10.1186/s13045-022-01359-4 36209111 PMC9548164

[B31] Malta T. M. Sokolov A. Gentles A. J. Burzykowski T. Poisson L. Weinstein J. N. (2018). Machine learning identifies stemness features associated with oncogenic dedifferentiation. Cell 173 (2), 338–354.e15. Epub 2018/04/07. 10.1016/j.cell.2018.03.034 29625051 PMC5902191

[B32] Michelakos T. Kontos F. Kurokawa T. Cai L. Sadagopan A. Krijgsman D. (2022). Differential role of hla-a and hla-B, C expression levels as prognostic markers in colon and rectal cancer. J. Immunother. cancer 10 (3), e004115. Epub 2022/03/13. 10.1136/jitc-2021-004115 35277460 PMC8919449

[B33] Miyamoto T. Murakami R. Hamanishi J. Tanigaki K. Hosoe Y. Mise N. (2022). B7-H3 suppresses antitumor immunity via the ccl2-ccr2-M2 macrophage Axis and contributes to ovarian cancer progression. Cancer Immunol. Res. 10 (1), 56–69. Epub 2021/11/21. 10.1158/2326-6066.Cir-21-0407 34799346 PMC9414298

[B34] Mollinedo F. (2019). Neutrophil degranulation, plasticity, and cancer metastasis. Trends Immunol. 40 (3), 228–242. Epub 2019/02/20. 10.1016/j.it.2019.01.006 30777721

[B35] Najafi M. Farhood B. Mortezaee K. (2019a). Cancer stem cells (cscs) in cancer progression and therapy. J. Cell Physiol. 234 (6), 8381–8395. 10.1002/jcp.27740 30417375

[B36] Najafi M. Hashemi Goradel N. Farhood B. Salehi E. Nashtaei M. S. Khanlarkhani N. (2019b). Macrophage polarity in cancer: a review. J. Cell. Biochem. 120 (3), 2756–2765. Epub 2018/10/03. 10.1002/jcb.27646 30270458

[B37] Nguyen L. W M. Martens J. Van Hoeck A. Cuppen E. (2020). Pan-cancer landscape of homologous recombination deficiency. Nat. Commun. 11 (1), 5584. 10.1038/s41467-020-19406-4 33149131 PMC7643118

[B38] Noblejas-López M. D. M. Nieto-Jiménez C. Morcillo García S. Pérez-Peña J. Nuncia-Cantarero M. Andrés-Pretel F. (2019). Expression of mhc class I, hla-a and hla-B identifies immune-activated breast tumors with favorable outcome. Oncoimmunology 8 (10), e1629780. 10.1080/2162402X.2019.1629780 31646075 PMC6791424

[B39] Pang S. Wang L. Wang S. Zhang Y. Wang X. (2020). Pesm: a novel approach of tumor purity estimation based on sample specific methylation sites. J. Bioinform Comput. Biol. 18 (5), 2050027. 10.1142/S0219720020500274 32757807

[B40] Park E.-G. Pyo S.-J. Cui Y. Yoon S.-H. Nam J.-W. (2022). Tumor immune microenvironment lncrnas. Brief. Bioinform 23 (1), bbab504. 10.1093/bib/bbab504 34891154 PMC8769899

[B41] Qin S. Long X. Zhao Q. Zhao W. (2020). Co-expression network analysis identified genes associated with cancer stem cell characteristics in lung squamous cell carcinoma. Cancer investig. 38 (1), 13–22. Epub 2019/11/27. 10.1080/07357907.2019.1697281 31770041

[B42] Reya T. Morrison S. J. Clarke M. F. Weissman I. L. (2001). Stem cells, cancer, and cancer stem cells. Nature 414 (6859), 105–111. Epub 2001/11/02. 10.1038/35102167 11689955

[B43] Shi Q. Xue C. Yuan X. He Y. Yu Z. (2020). Gene signatures and prognostic values of m1a-related regulatory genes in hepatocellular carcinoma. Sci. Rep. 10 (1), 15083. Epub 2020/09/17. 10.1038/s41598-020-72178-1 32934298 PMC7492257

[B44] Shi X. Liu Y. Cheng S. Hu H. Zhang J. Wei M. (2021). Cancer stemness associated with prognosis and the efficacy of immunotherapy in adrenocortical carcinoma. Front. Oncol. 11, 651622. 10.3389/fonc.2021.651622 34367952 PMC8334864

[B45] Song P. Tayier S. Cai Z. Jia G. (2021). Rna methylation in mammalian development and cancer. Cell Biol. Toxicol. 37 (6), 811–831. 10.1007/s10565-021-09627-8 34272618 PMC8599391

[B46] Sun Y. Ling C. (2019). Analysis of the long non-coding rna Linc01614 in non-small cell lung cancer. Med. Baltim. 98 (30), e16437. Epub 2019/07/28. 10.1097/MD.0000000000016437 PMC6708815 31348244

[B47] Sung H. Ferlay J. Siegel R. L. Laversanne M. Soerjomataram I. Jemal A. (2021). Global cancer statistics 2020: globocan estimates of incidence and mortality worldwide for 36 cancers in 185 countries. CA Cancer J. Clin. 71 (3), 209–249. 10.3322/caac.21660 33538338

[B48] Veglia F. Gabrilovich D. I. (2017). Dendritic cells in cancer: the role revisited. Curr. Opin. Immunol. 45, 43–51. Epub 2017/02/14. 10.1016/j.coi.2017.01.002 28192720 PMC5449252

[B49] Vishnubalaji R. Shaath H. Elkord E. Alajez N. M. (2019). Long non-coding RNA (lncRNA) transcriptional landscape in breast cancer identifies LINC01614 as non-favorable prognostic biomarker regulated by TGFβ and focal adhesion kinase (FAK) signaling. Cell Death Discov. 5, 109. Epub 2019/07/03. 10.1038/s41420-019-0190-6 31263577 PMC6591245

[B50] Wan Q. Ren X. Tang J. Ma K. Deng Y.-P. (2023). Cross talk between tumor stemness and microenvironment for prognosis and immunotherapy of uveal melanoma. J. Cancer Res. Clin. Oncol. 149 (13), 11951–11968. 10.1007/s00432-023-05061-x 37420017 PMC11797443

[B51] Wang B. Niu L. Wang Z. Zhao Z. (2021c). Rna M1a methyltransferase Trmt6 predicts poorer prognosis and promotes malignant behavior in glioma. Front. Mol. Biosci. 8, 692130. Epub 2021/10/12. 10.3389/fmolb.2021.692130 34631793 PMC8493077

[B52] Wang C. Li Y. Jia L. Kim J. K. Li J. Deng P. (2021a). Cd276 expression enables squamous cell carcinoma stem cells to evade immune surveillance. Cell stem Cell 28 (9), 1597–1613.e7. Epub 2021/05/05. 10.1016/j.stem.2021.04.011 33945793 PMC8419062

[B53] Wang D. Zhang H. Fang X. Cao D. Liu H. (2020a). Pan-cancer analysis reveals the role of long non-coding rna Linc01614 as a highly cancer-dependent oncogene and biomarker. Oncol. Lett. 20 (2), 1383–1399. Epub 2020/07/30. 10.3892/ol.2020.11648 32724381 PMC7377058

[B54] Wang D. R. Wu X. L. Sun Y. L. (2022a). Therapeutic targets and biomarkers of tumor immunotherapy: response versus non-response. Signal Transduct. Target Ther. 7 (1), 331. Epub 2022/09/20. 10.1038/s41392-022-01136-2 36123348 PMC9485144

[B55] Wang H. Wu J. Guo W. (2020b). Sp1-Mediated upregulation of lncrna Linc01614 functions a cerna for mir-383 to facilitate glioma progression through regulation of Adam12. Onco Targets Ther. 13, 4305–4318. Epub 2020/06/18. 10.2147/OTT.S242854 32547064 PMC7244248

[B56] Wang J. Z. Zhu W. Han J. Yang X. Zhou R. Lu H. C. (2021b). The role of the hif-1α/alyref/pkm2 Axis in glycolysis and tumorigenesis of bladder cancer. Cancer Commun. Lond. Engl. 41 (7), 560–575. Epub 2021/05/16. 10.1002/cac2.12158 PMC8286140 33991457

[B57] Wang L. Zhang J. Su Y. Maimaitiyiming Y. Yang S. Shen Z. (2022b). Distinct roles of M(5)C rna methyltransferase Nsun2 in major gynecologic cancers. Front. Oncol. 12, 786266. Epub 2022/03/15. 10.3389/fonc.2022.786266 35280737 PMC8916577

[B58] Wang Y. Song B. Zhu L. Zhang X. (2019a). Long non-coding rna, Linc01614 as a potential biomarker for prognostic prediction in breast cancer. PeerJ 7, e7976. Epub 2019/11/20. 10.7717/peerj.7976 31741788 PMC6858983

[B59] Wang Y. Zeng L. Liang C. Zan R. Ji W. Zhang Z. (2019b). Integrated analysis of transcriptome-wide M(6)a methylome of osteosarcoma stem cells enriched by chemotherapy. Epigenomics 11 (15), 1693–1715. Epub 2019/10/28. 10.2217/epi-2019-0262 31650864

[B60] Wei W. Wei X. Xie X. (2023). Linc01614 regulates the proliferation, apoptosis, and chemotherapy resistance in esophageal squamous cell carcinoma by targeting mir-4775. Iran. J. Public Health 52 (6), 1170–1180. Epub 2023/07/24. 10.18502/ijph.v52i6.12959 37484148 PMC10362831

[B61] Woo H. H. Chambers S. K. (2019). Human alkbh3-induced M(1)a demethylation increases the csf-1 mrna stability in breast and ovarian cancer cells. Biochimica biophysica acta Gene Regul. Mech. 1862 (1), 35–46. Epub 2018/10/21. 10.1016/j.bbagrm.2018.10.008 30342176

[B62] Wu H. Zhou J. Chen S. Zhu L. Jiang M. Liu A. (2021). Survival-related lncrna landscape analysis identifies Linc01614 as an oncogenic lncrna in gastric cancer. Front. Genet. 12, 698947. Epub 2021/10/26. 10.3389/fgene.2021.698947 34691143 PMC8526963

[B63] Xu H. H. Wang H. L. Xing T. J. Wang X. Q. (2022). A novel prognostic risk model for cervical cancer based on immune checkpoint hla-G-driven differentially expressed genes. Front. Immunol. 13, 851622. Epub 2022/08/05. 10.3389/fimmu.2022.851622 35924232 PMC9341272

[B64] Yan H. Bu P. (2021). Non-coding rna in cancer. Essays Biochem. 65 (4), 625–639. Epub 2021/04/17. 10.1042/ebc20200032 33860799 PMC8564738

[B65] Yan S. Xu J. Liu B. Ma L. Tan H. Fang C. (2021). Integrative bioinformatics analysis identifies Linc01614 as a potential prognostic signature in esophageal cancer. Transl. Cancer Res. 10 (4), 1804–1812. 10.21037/tcr-20-2529 35116503 PMC8798299

[B66] Zhang B. Zhang X. Zhou T. Liu J. (2015). Clinical observation of liver cancer patients treated with Axitinib and cabozantinib after failed sorafenib treatment: a case report and literature review. Cancer Biol. Ther. 16 (2), 215–218. Epub 2015/02/11. 10.4161/15384047.2014.962318 25668362 PMC4622678

[B67] Zhang Z. Zhang C. Luo Y. Zhang G. Wu P. Sun N. (2021). Rna N(6) -methyladenosine modification in the lethal teamwork of cancer stem cells and the tumor immune microenvironment: current landscape and therapeutic potential. Clin. Transl. Med. 11 (9), e525. Epub 2021/09/30. 10.1002/ctm2.525 34586737 PMC8473646

[B68] Zhao M. Chen Z. Zheng Y. Liang J. Hu Z. Bian Y. (2020). Identification of cancer stem cell-related biomarkers in lung adenocarcinoma by stemness index and weighted correlation network analysis. J. cancer Res. Clin. Oncol. 146 (6), 1463–1472. Epub 2020/03/30. 10.1007/s00432-020-03194-x 32221746 PMC11804350

