# Supplementary figures and images for: LINC01614 is a promising diagnostic and prognostic marker in HNSC linked to the tumor microenvironment and oncogenic function
Source: Front Genet. 2024 Apr 9;15:1337525. doi: 10.3389/fgene.2024.1337525 (PMC11035733; doi:10.3389/fgene.2024.1337525)

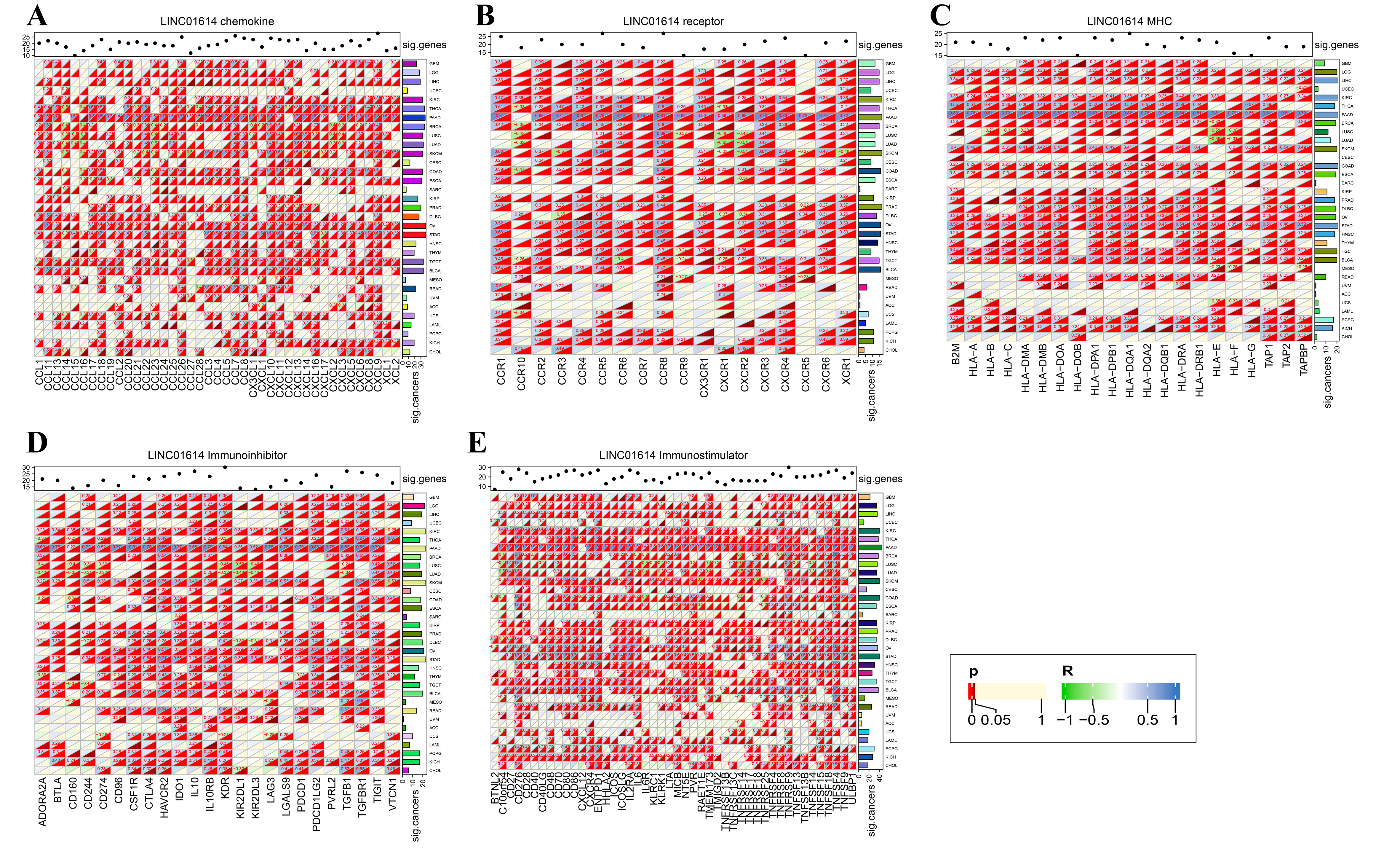

Supplement: Supplementary file 1 [file Image3.TIF]

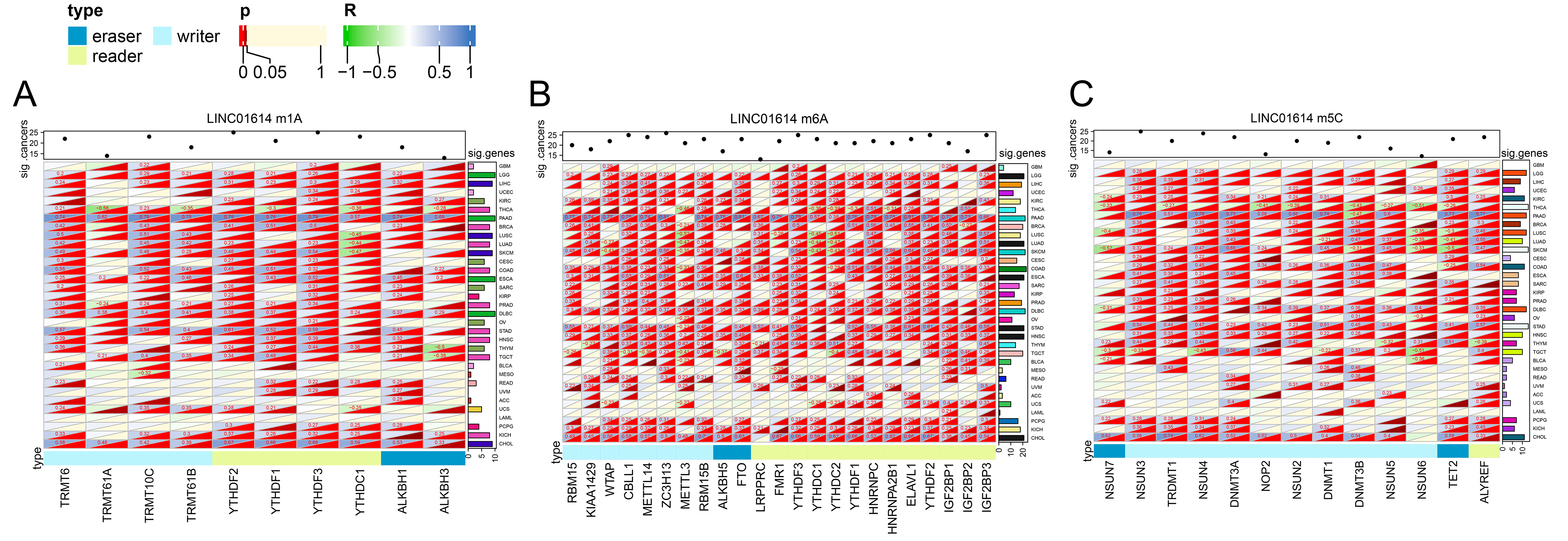

Supplement: Supplementary file 2 [file Image4.TIF]

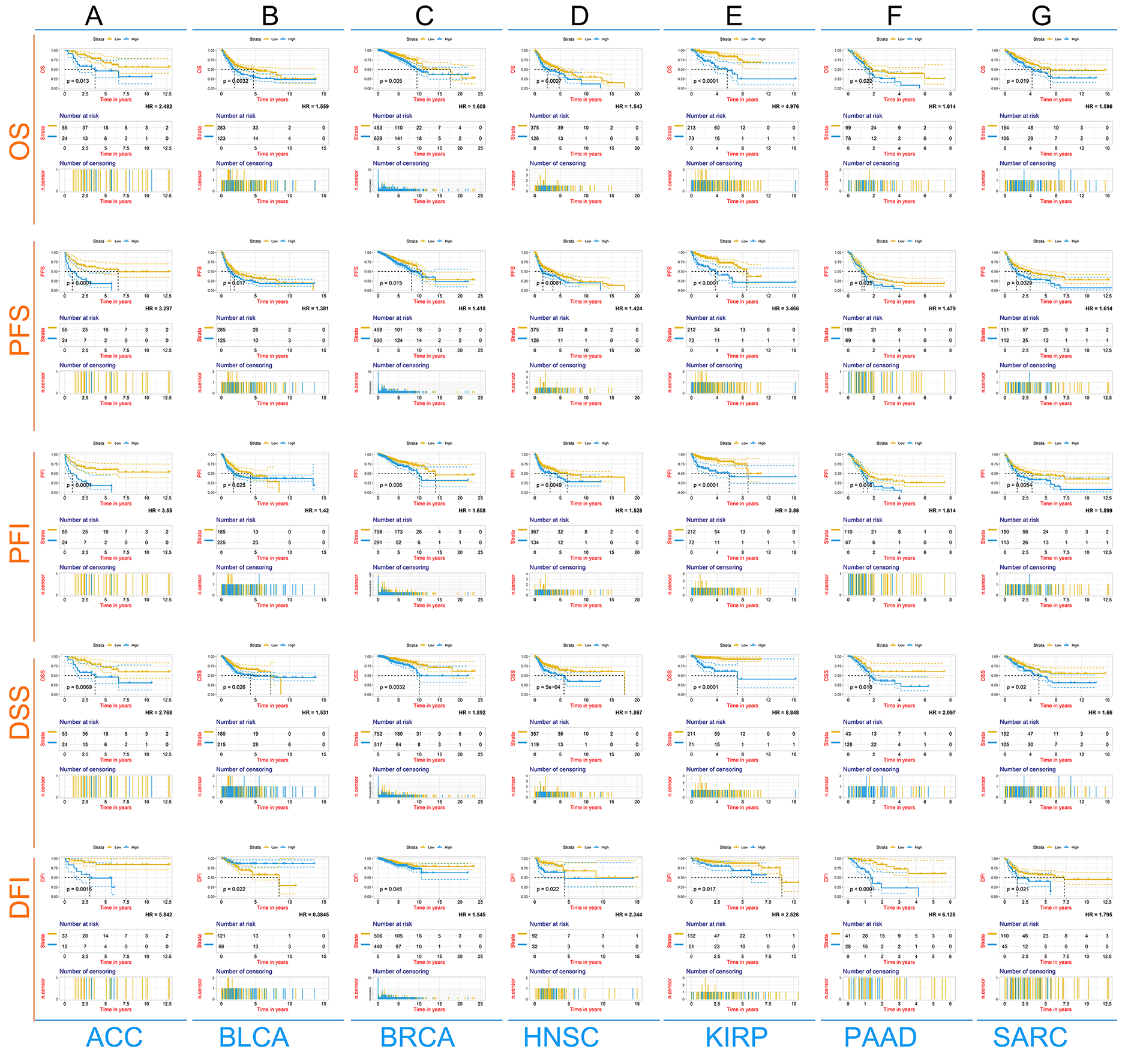

Supplement: Supplementary file 4 [file Image2.TIF]

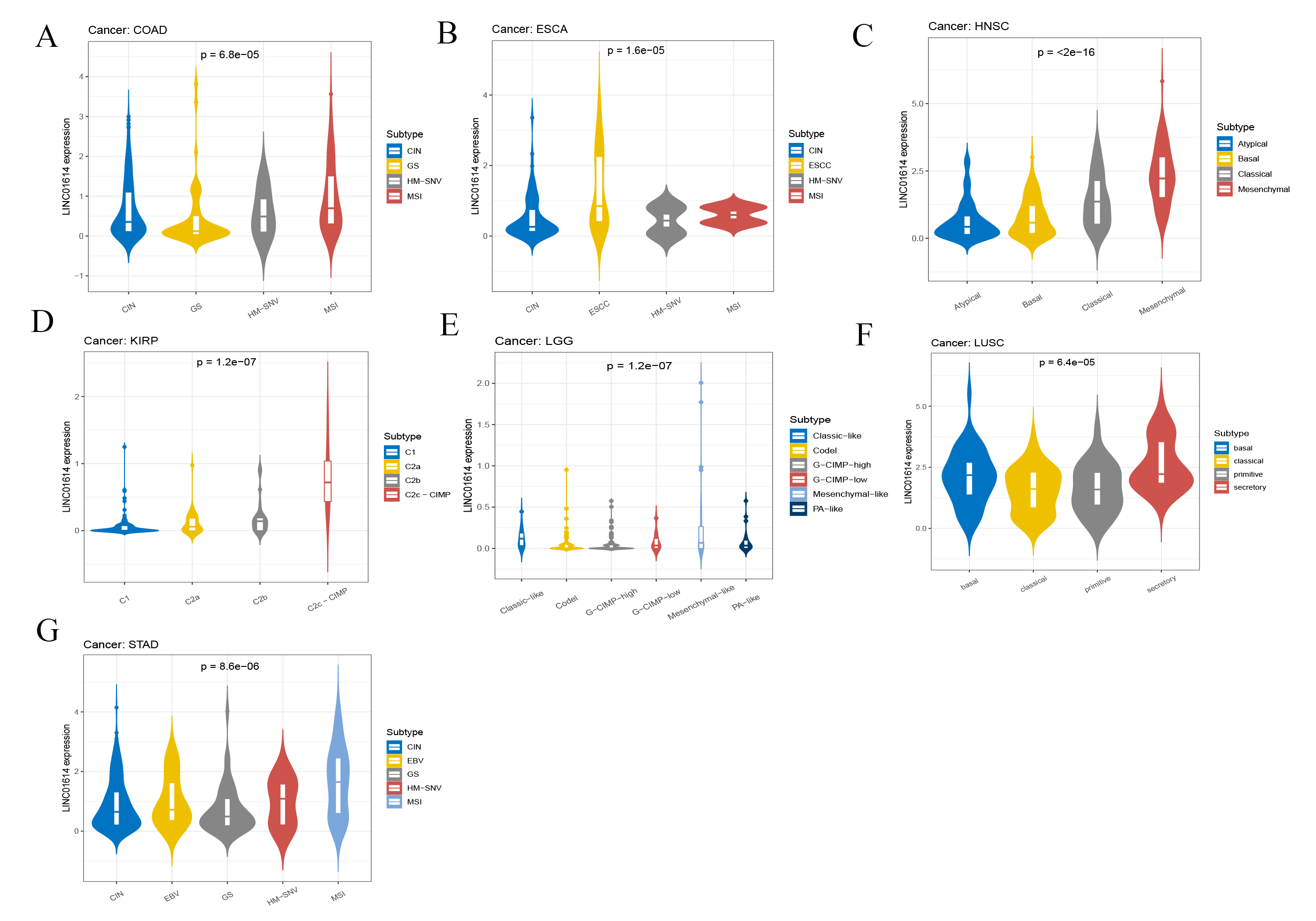

Supplement: Supplementary file 5 [file Image1.TIF]

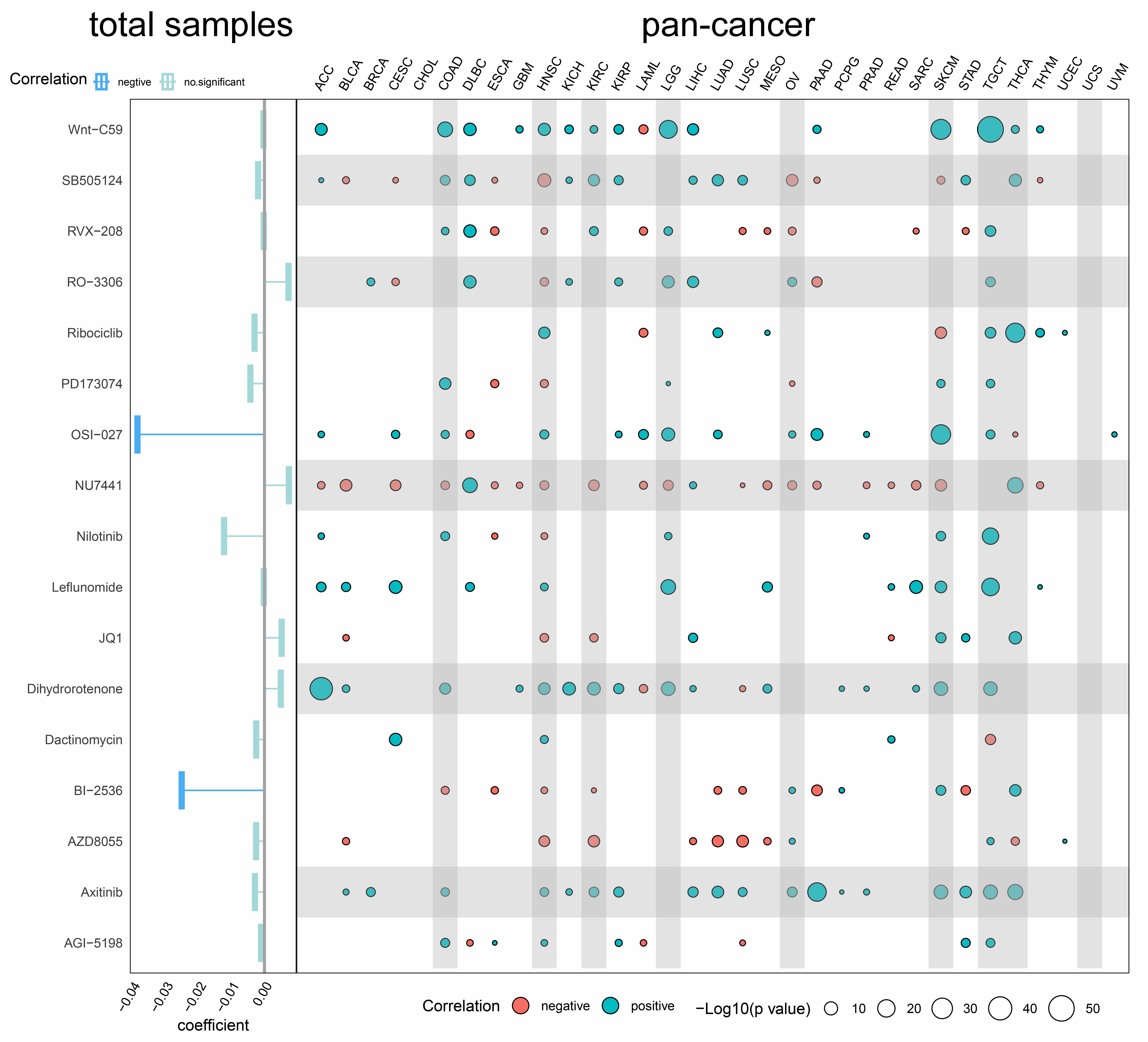

Supplement: Supplementary file 7 [file Image5.TIF]
