# Supplementary material for: LINC01614 is a promising diagnostic and prognostic marker in HNSC linked to the tumor microenvironment and oncogenic function
Source: Front Genet. 2024 Apr 9;15:1337525. doi: 10.3389/fgene.2024.1337525 (PMC11035733; doi:10.3389/fgene.2024.1337525)
Supplement: Supplementary file 6 [file Table2.DOCX]

Supplementary Material

**Supplementary Information**

**Table S1.** The significant enriched HALLMARK pathways of LINC01614 in HNSC by GSEA.

**Table S2.** The 101 genes that significantly coexpressed with LINC01614 and associated with lymph node metastasis in HNSC.

**Table S3.** The significant enriched GO and KEGG pathways of LINC01614 coexpressed and lymph node metastasis associated genes in HNSC by “clusterProfiler” package.

**Table S4.** The detailed list of PPI of linc01614 coexpressed genes obtained through the STRING database.

**Table S5.** The 193 miRNAs that significantly associated with LINC01614 co-expressed genes illustrated by miRNA screen process.

**Table S6.** The miRNAs that predicted as targets of LINC01614.

**Figure S1.** Significant correlations of LINC01614 expression with molecular subtypes. (A) COAD, (B) ESCA, (C) HNSC, (D) KIRP, (E) LGG, (F) LUSC, (G) STAD.

**Figure S2.** Kaplan-Meier curves of LINC01614 expression level in different cancers. Kaplan-Meier curves of the five survival endtime points (OS, PFS, PFI, DSS and PFI) for LINC01614 expression level in the selected 7 significant cancers including (A) ACC, (B) BLCA, (C) BRCA, (D) HNSC, (E) KIRP, (F) PAAD and (G) SARC.

**Figure S3.** Coexpression of LINC01614 with various immune-related genes. The Spearman correlation analysis was performed to assess the relationship between the expression of LINC01614 and immune-related genes, which encompassed (A) chemokines, (B) chemokine receptors, (C) immune stimulators, (D) immune inhibitors, and (E) MHCs.

**Figure S4.** The coexpression of LINC01614 and RNA methylation related genes. (A) The Spearman correlation analyzed the relationship between the expression of LINC01614 and m1A related genes. (B) The Spearman correlation investigated the relationship between the expression of LINC01614 and m6A related genes. (C) The Spearman correlation explored the relationship between the expression of LINC01614 and m5C related genes.

**Figure S5.** The relationship between LINC01614 expression and drug sensitivity.
